# Comparative analyses of American and Asian lotus genomes reveal insights into petal color, carpel thermogenesis and domestication

**DOI:** 10.1111/tpj.15753

**Published:** 2022-04-19

**Authors:** Ping Zheng, Heng Sun, Juan Liu, Jishan Lin, Xingtan Zhang, Yuan Qin, Wenping Zhang, Xiuming Xu, Xianbao Deng, Dong Yang, Meng Wang, Yanting Zhang, Heyun Song, Yongji Huang, Warner Orozco‐Obando, Ray Ming, Mei Yang

**Affiliations:** ^1^ Center for Genomics and Biotechnology, Fujian Provincial Key Laboratory of Haixia Applied Plant Systems Biology, Key Laboratory of Genetics, Breeding and Multiple Utilization of Corps Ministry of Education, Fujian Agriculture and Forestry University Fuzhou 350002 Fujian China; ^2^ Key Laboratory of Plant Germplasm Enhancement and Specialty Agriculture Wuhan Botanical Garden, Chinese Academy of Sciences Wuhan 430074 China; ^3^ Center of Economic Botany Core Botanical Gardens, Chinese Academy of Sciences Wuhan 430074 China; ^4^ Virginia Cooperative of Extension Virginia Polytechnic Institute and State University Blacksburg VA 24061 USA; ^5^ Department of Plant Biology University of Illinois at Urbana‐Champaign Urbana IL 61801 USA

**Keywords:** lotus, genome sequencing, genetic divergence, petal color, carpel thermogenesis, domestication

## Abstract

*Nelumbo lutea* (American lotus), which differs from *Nelumbo nucifera* (Asian lotus) morphologically, is one of the two remaining species in the basal eudicot family Nelumbonaceae. Here, we assembled the 843‐Mb genome of American lotus into eight pseudochromosomes containing 31 382 protein‐coding genes. Comparative analyses revealed conserved synteny without large chromosomal rearrangements between the genomes of American and Asian lotus and identified 29 533 structural variants (SVs). Carotenoid and anthocyanin pigments determine the yellow and red petal colors of American and Asian lotus, respectively. The structural genes encoding enzymes of the carotenoid and anthocyanin biosynthesis pathways were conserved between two species but differed in expression. We detected SVs caused by repetitive sequence expansion or contraction among the anthocyanin biosynthesis regulatory *MYB* genes. Further transient overexpression of candidate *NnMYB5* induced anthocyanin accumulation in lotus petals. Alternative oxidase (AOX), uncoupling proteins (UCPs), and sugar metabolism and transportation contributed to carpel thermogenesis. Carpels produce heat with sugars transported from leaves as the main substrates, because there was weak tonoplast sugar transporter (TST) activity, and with *SWEET*s were highly expressed during thermogenesis. Cell proliferation‐related activities were particularly enhanced in the warmer carpels compared with stamens during the cold night before blooming, which suggested that thermogenesis plays an important role in flower protogyny. Population genomic analyses revealed deep divergence between American and Asian lotus, and independent domestication affecting seed, rhizome, and flower traits. Our findings provide a high‐quality reference genome of American lotus for exploring the genetic divergence and variation between two species and revealed possible genomic bases for petal color, carpel thermogenesis and domestication in lotus.

## Significance Statement

A chromosome‐level genome assembly of American lotus was generated to examine the divergence of its genome from that of Asian lotus, and to reveal the genomic bases of petal color, carpel thermogenesis and domestication in these species.

## INTRODUCTION

*Nelumbo* is the only genus in the plant family Nelumbonaceae, and comprises two extant species: *Nelumbo lutea* Willd. and *Nelumbo nucifera* Gaertn. (Lin et al., 2019). The American lotus, *N. lutea*, separated from the Asian lotus, *N. nucifera*, after the extinction of intermediate types during the Ice Age. These two species exhibit differences in morphology, such as plant size, leaf shape, petal shape and petal color, but share the same chromosome number (2*n* = 16). Crosses between them can generate viable progeny, indicating no complete interspecific reproductive barrier. Asian lotus was domesticated as a vegetable and ornamental crop, with three specialized groups of cultivars specifically for flower, seed or rhizome crops (Yang et al., 2015). Flowering lotus exhibits ornamental flowers with attractive flower shapes and petal colors. Seed lotus cultivars produce a higher yield of seeds, and rhizome lotus cultivars produce an enlarged rhizome and relatively fewer flowers (Lin et al., 2019). American lotus is wild and has been used widely for breeding ornamental lotus through interspecific hybridization.

Petal color is the most obvious difference between the two species of *Nelumbo*. American lotus bears yellow flowers whereas wild Asian lotus produces red flowers. Ornamental flowering lotus cultivars derived from interspecific or intraspecific crosses exhibit a variety of petal colors, including red, pink, white with red borders, plain white and yellow (Lin et al., 2019). The red pigments in Asian lotus have been characterized as five anthocyanins, including delphinidin 3‐*O*‐glucoside, cyanidin 3‐*O*‐glucoside, petunidin 3‐*O*‐glucoside, peonidin 3‐*O*‐glucoside and malvidin 3‐*O*‐glucoside (Yang et al., 2009). However, anthocyanins are not detected in the yellow flowers of American lotus, the colors of which are determined by non‐anthocyanin flavonoids or carotenoids (Sun et al., 2016). The biosynthesis pathways of anthocyanin and carotenoid are conserved and have been well characterized in plants (Tanaka et al., 2008). Certain *MYB* genes encode the key factors that have activating or suppressive effects on the transcript expression of the genes that encode the biosynthetic enzyme in both pathways (Chiu et al., 2010; Quattrocchio et al., 1999; Sagawa et al., 2016). The structural genes and *MYB*s related to anthocyanin biosynthesis have been identified in red‐petaled lotus (Deng et al., 2021; Sun et al., 2016), but candidate genes responsible for the differentiation of flower color between the two species remain unclear.

The lotus flower is protogynous and can maintain a constant temperature of 30–36°C during anthesis for 2–4 days, despite fluctuations in ambient temperature (Seymour & Schultze‐Motel, 1998; Watling et al., 2006). This period of thermogenesis in lotus is associated with the developmental sequence of the flowers (Seymour & Schultze‐Motel, 1998). Flower thermogenesis reaches its peak on the first day of blooming. At this time the petals open partially, providing access for insect pollinators, and the carpels mature while the stamens remain immature. Subsequently, the stamens mature and the flowers open more widely on the next day. Then, the petals and stamens abscise, and thermogenesis can no longer be detected. The floral receptacle is reportedly the main organ driving the evolution of heat, using starch as fuel during the thermogenic stage (Grant et al., 2008; Miller et al., 2009; Watling et al., 2006). Two energy‐dissipating systems, alternative oxidase (AOX) and uncoupling protein (UCP), are thought to be of great importance in plant heat generation. AOX is responsible for controlling lotus thermogenesis (Sun et al., 2021; Watling et al., 2006). Although UCPs are co‐expressed with AOX during thermogenesis, their physiological roles during thermogenesis in lotus have not yet been clarified. Thermogenesis provides the optimal temperature for floral growth and reproduction, and attracts insect pollinators through heat rewards or through the volatilization of scents (Breidenbach et al., 1997; Li & Huang, 2009; Seymour & Matthews, 2006; Terry et al., 2004; Yafuso, 1993). However, the function and genomic basis of thermogenesis in lotus remain unclear.

Two genomes of Asian lotus have previously been assembled and anchored into pseudochromosomes (Gui et al., 2018; Ming et al., 2013; Shi et al., 2020; Wang et al., 2013). To explore the genomic divergence of, and diversity between, American and Asian lotus, we sequenced the genome of American lotus, analyzed its genomic structure, and identified variants between its genome and that of Asian lotus. Through our comparative genomics of these two lotus species, we explored the molecular bases for petal color and carpel thermogenesis. Moreover, we collected 240 lotus accessions worldwide and resequenced their genomes to analyze their genetic divergence, population structure and selective sweeps.

## RESULTS

### Genome sequencing and analyses of American lotus

**Figure 1:**
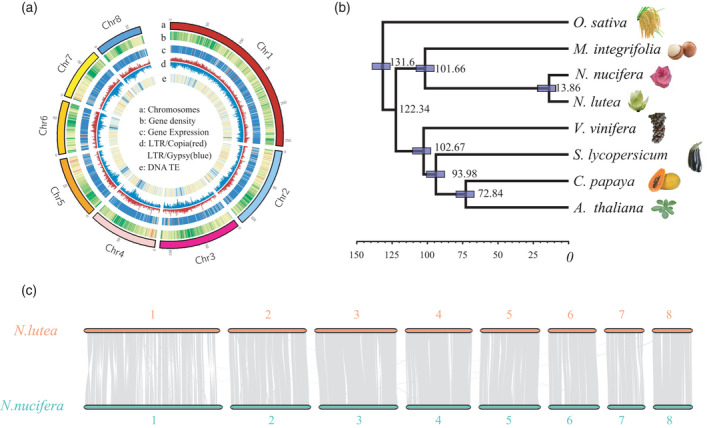
Characteristics of the *Nelumbo lutea* (American lotus) genome. (a) Distribution of genomic features along the American lotus genome. The rings indicate (from outermost to innermost): a, the karyotype in Mb; b, gene density; c, gene transcript expression; d, long terminal repeats (LTRs); and e, DNA transposable elements (TEs). Dark red represents higher gene density, higher gene transcript expression or higher DNA TE content. (b) Inferred phylogenetic tree across eight plant species. Divergence times between *Arabidopsis thaliana* and *Carica papaya* (68–72 Mya) and between monocots and eudicots (120–140 Mya) are used as calibrators. (c) Synteny blocks between American lotus (*Nelumbo lutea* Willd.) and Asian lotus (*Nelumbo nucifera* Gaertn.). Genome synteny analysis suggests highly conserved karyotypes between the two lotus species. Synteny analysis was performed using mcscan and genes were visualized in blocks of 10. [Colour figure can be viewed at wileyonlinelibrary.com]

The genome of American lotus, *N. lutea*, was assembled from sequence information obtained using PacBio RSII sequencing technology and high‐throughput chromatin conformation capture (Hi‐C) (Table S1). We generated 74.6 Gb reads with 79× depth of the estimated 944‐Mb American lotus genome (Diao et al., 2006). The genome was assembled into 1652 contigs with a total length of 843 Mb and an N50 length (the sequence length of the shortest contig at 50% of the total genome length) of 1.34 Mb (Table S2). A total of 50.32 Gb of Hi‐C data were generated for chromosome architecture mapping. Finally, 1597 contigs with a total length of 839 Mb were anchored into eight pseudochromosomes, accounting for 99.44% of the total assembly (Figure 1a; Table S3). The concentrated interaction patterns shown as an Hi‐C heat map and the 99.99% coverage rate of RNA sequencing (RNA‐seq) reads support this well‐organized chromosomal‐level assembly (Figure S1; Table S4). The large genome size of American lotus might be attributable to its high DNA repeat content. The total of 680 Mb of repetitive sequences in the American lotus genome that we identified, among which retrotransposons are the most abundant (49.0%, with more Ty1/Copia than Ty3/Gypsy long terminal repeats, account for 81.0% of the assembled genome; Figures 1a and S2; Table S5). A total of 31 382 high‐confidence protein‐coding genes were annotated in the American lotus genome. BUSCO analysis indicated that 90.7% of the core conserved plant genes were complete (Table S6) in the genome of this species.

We further performed comparative genomic analyses to identify orthologous groups in the genomes of *N. lutea* and five other species, including *Arabidopsis thaliana*, *Carica papaya*, *N. nucifera, Oryza sativa* and *Vitis vinifera*. A maximum‐likelihood phylogenic tree from 924 single‐copy orthologs showed that the divergence time between *N. lutea* and *N. nucifera* was about 13.86 million years ago (13.86 Mya) (Figure 1b). All genes from these six species clustered into 15 685 groups and 56.5% (8859) of them were shared among the species (Figure S3; Table S7). In *N. lutea*, 83.34% of all predicted proteins clustered into 14 167 groups, and 67 proteins from 13 groups were species specific (Figure S4; Table S8). Because *N. nucifera* and *N. lutea* derive from same ancestor, the identification of the genes specific to each species could help to reveal the genetic reasons for any unique physiological characteristics or ecological adaptations. Compared with *N. lutea*, 501 genes were specifically identified in *N. nucifera*, and Gene Ontology (GO) enrichment analyses indicated that these genes were over‐represented for the molecular function of ‘catalytic activities’ (Tables S9 and S10). Further, 38 genes were specifically identified in *N. lutea*, most of which were previously unknown genes without matches in the non‐redundant (nr) database of the National Center for Biotechnology Information (NCBI) (Table S9).

### Comparative analyses of the genomes of American and Asian lotus

Although the size of the American lotus genome is 30 Mb larger than that of the Asian lotus, their genomic sequences displayed good synteny and collinearity and we did not detect any large chromosomal rearrangements (Figure 1c). Approximately 92.4% of the American lotus genes show a one‐to‐one synteny pattern with 88.5% of the Asian lotus genes. Direct genomic alignment detected 29 533 structural variants (SVs) with a total length of 36.46 Mb between American and Asian lotus (Table S11). Based on the spacing and orientation of the alignments, we classified the SVs into six groups, including 9160 insertions, 5732 deletions, 711 tandem expansions, 56 tandem contractions, 6964 repeat expansions and 6910 repeat contractions. The detected SVs exhibit a broad size distribution, ranging from 50 to 10 000 bp. Insertions and deletions (InDels) are the main types of SV between these two species, and small ones (50–500 bp) account for approximately 90% of these InDels. In contrast, most of the repeat variants are large and range from 500 to 10 000 bp (Figure S5). Variants taking place within tandem sequences represented the least abundant SVs. Tandem expansions are more common than tandem contractions in American lotus, which might explain the larger genome size of American lotus compared with Asian lotus (Figure S6).

We then analyzed the distribution of SVs across the American lotus genome and found that most SVs were located in non‐coding genomic regions (Table S12). A total of 62.46% of the detected SVs appear in intergenic regions and 23.03% appear in intronic regions. A total of 6093 genes contain SVs in their genic regions, including 1260 (4.26%) SVs in exonic regions. GO enrichment analyses were performed to analyze the possible biological functions and pathways of SV‐associated genes. We found that the SV‐associated genes were over‐represented in terms such as ‘regulation of mitotic cell cycle’, ‘embryo development ending in seed dormancy’ and ‘protein transporter activity’ (Table S13).

### Petal color differentiation in American and Asian lotus

**Figure 2:**
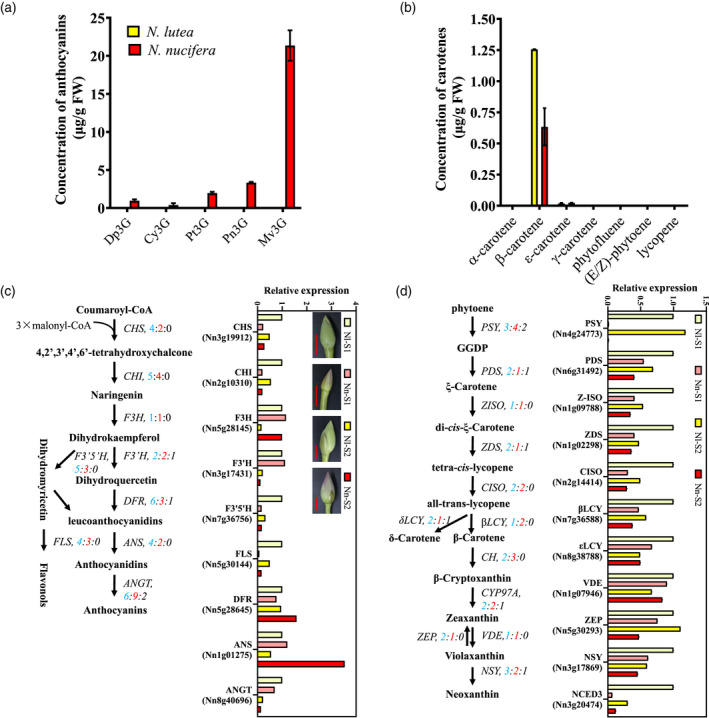
Anthocyanin and carotenoid pigments in petals of American and Asian lotus flowers. (a) Concentrations of five anthocyanins in petals. (b) Concentration of carotenoids in petals. (c) Diagram of the anthocyanin biosynthesis pathway and the relative expression of transcripts from its structural genes in *Nelumbo lutea* and *Nelumbo nucifera*. (d) Diagram of the carotenoid biosynthesis pathway and the relative expression of transcripts from its structural genes in *N. lutea* and *N. nucifera*. In (c) and (d), gene numbers in *N. lutea* (blue), *N. nucifera* (red) and structurally variant (SV) regions (black) are shown for each pathway. qRT‐PCR was used to measure the relative transcript expression of pigment pathway genes in *N. lutea* and *N. nucifera* petals at different developmental stages: Nl–S1, Nl–S2, Nn–S1 and Nn–S2 describe petals sampled from *N. lutea* (Nl) and *N. nucifera* (Nn) flowerbuds at the developmental stages S1 and S2, respectively. S1, the length of flowerbud is about 2–3 cm, S2 the length of flowerbud is about 5–6 cm. Scale bars in (c): 2 cm. [Colour figure can be viewed at wileyonlinelibrary.com]

Anthocyanins are the pigments responsible for the red color of Asian lotus petals. We detected five glucoside anthocyanins in the petals of Asian lotus ‘China Antique’, with a total concentration of 27.05 ± 2.52 μg g^−1^ FW. The major compound, Mv‐3‐glu, accounted for 76% of the total anthocyanin content in petals. No anthocyanins were detected in the petals from American lotus (Figure 2a). The concentration of non‐anthocyanin flavonoids, such as flavones or flavanols, was significantly higher in American lotus petals (576.41 ± 23.54 μg g^−1^ FW) than in Asian lotus petals (136.67 ± 10.82 μg g^−1^ FW). Specifically, quercetins accounted for the majority of flavonoids in American lotus petals, whereas kaempferol derivatives accounted for the majority of flavonoids in Asian lotus petals (Figure S7). In addition to flavonoids, carotenoids were also responsible for the yellow color of American lotus petals. Compared with Asian lotus, the total content of carotenoids was higher in the yellow petal of the American lotus. A total of 18 carotenoids were identified in the petals of two species, including two carotenes (β‐carotene and ε‐carotene) and 16 xanthophylls (Figures 2b and S7). The contents of β‐carotene and several xanthophylls were higher in American lotus than in Asian lotus. Moreover, β‐cryptoxanthin palmitate and rubixanthin palmitate were only detected in American lotus (Figure S7).

The genes encoding enzymes that mediate anthocyanin biosynthesis, including *anthocyanin 3‐O‐glucosyltransferase* (*ANGT*), *anthocyanidin synthase* (*ANS*), *chalcone isomerase* (*CHI*), *chalcone synthase* (*CHS*), *dihydroflavonol 4‐reductase* (*DFR*), *flavanone 3‐hydroxylase* (*F3H*), *flavonoid 3′‐hydroxylase* (*F3′H*) and *flavonoid 3′5′‐hydroxylase* (*F3′5′H*), were identified in both two species Figure 2c; Table S14). Gene copy numbers of early biosynthesis genes such as *F3H* and *F3′H* were the same in American and Asian lotus. Transcript expression of *F3H* and *F3′H* were higher in American lotus petals than in Asian lotus petals (Figure 2c). We observed tandem duplications of late biosynthesis genes, including *DFR*, *ANS* and *ANGT*, which are key genes leading to anthocyanin biosynthesis in American or Asian lotus. These three genes exhibited higher transcript expression in Asian lotus than in American lotus (Figure 2c). SVs were detected in *F3′H*, *DFR* or *ANGT*, and the SVs in *F3′H* (*Nn3g17431*) and *ANGT* (*Nn7g36416*) were located within their exons. SVs also were detected in six key genes in the carotenoid biosynthesis pathway, but only one was located within an exon of *phytoene synthase* (*PSY*, *Nn7g38019*) (Figure 2d; Tables S15 and S16). All of the carotenoid biosynthesis genes, especially the *PSY* gene, which conducts the rate‐limiting step of carotenoid biosynthesis, exhibited higher expression in American lotus petals (Figure 2d).

**Figure 3:**
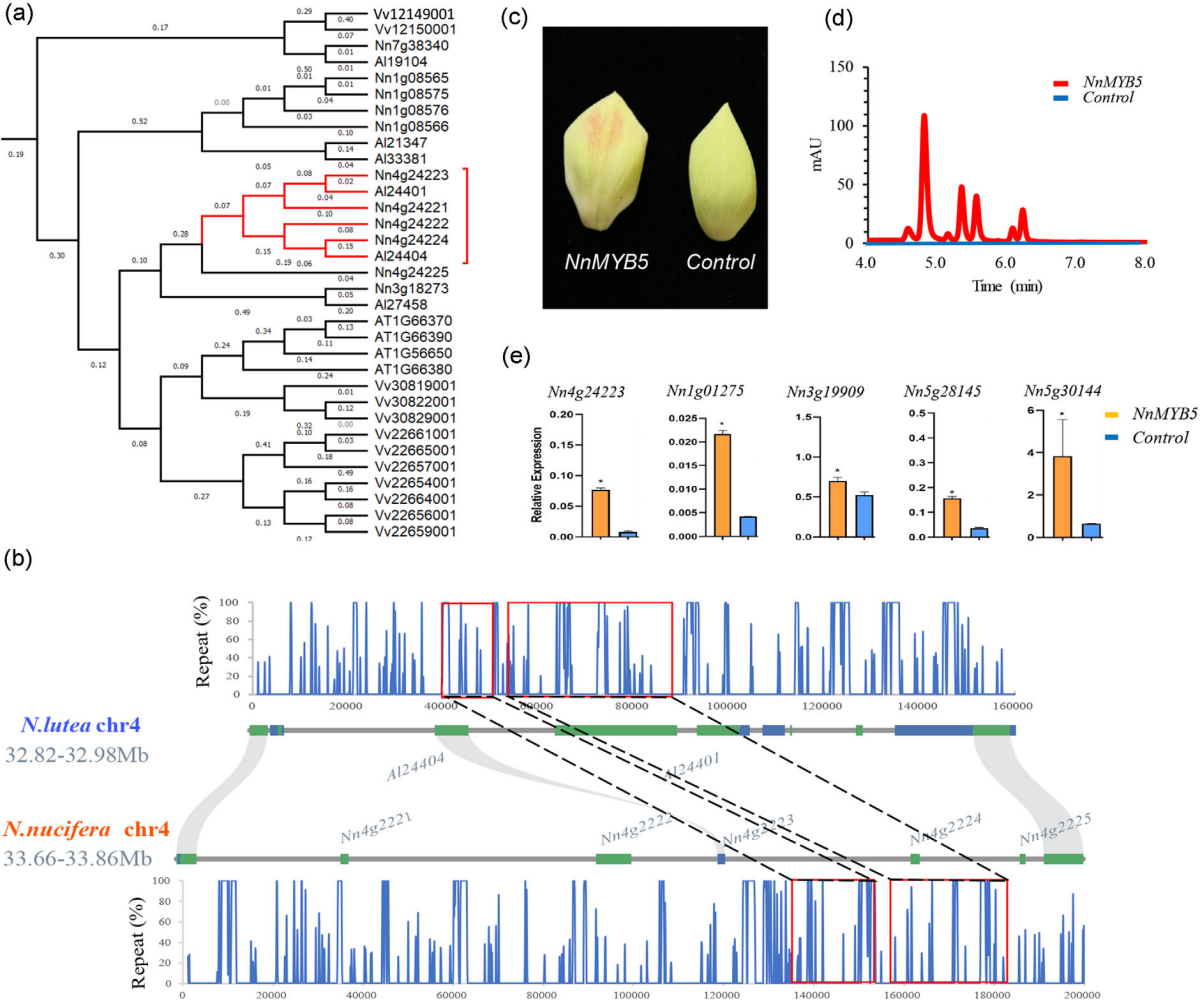
Comparison of pigment pathway regulatory *MYB* genes in American and Asian lotus. (a) Phylogenetic tree of TT2 clade flavonoid biosynthesis regulatory *MYB*s. Gene expansions in TT2 clade *MYB*s detected in Asian lotus, but not in American lotus, are highlight in red. (b) Structural variants between American and Asian lotus in genomic sequences of members (*Nn4g24221*, *Nn4g24222*, *Nn4g24223*, *Nn4g24224* and *Nn4g24225*) of an *MYB* gene cluster on chromosome 4. The middle panel shows the synteny of *NnMYB* genomic regions. The upper and lower panels show DNA repeat contents in 1000‐bp windows with 100‐bp steps. Two large structural variants are highlighted in red boxes. The first represents a repeat contraction, whereas the second represents a repeat expansion in American lotus. (c) Transient expression of the *NnMYB5* (*Nn4g24223*) gene in white petals of Asian lotus could induce anthocyanin accumulation. (d) HPLC chromatograph of anthocyanin profile upon transient overexpression of *NnMYB5* (*Nn4g24223*) in lotus petals. (e) Relative expression of anthocyanin biosynthesis genes upon transient overexpression of *NnMYB5* in lotus petals. Increased relative transcript expression of *NnANS* (*Nn1g01275*), *NnCHS* (*Nn3g19909*), *NnF3H* (*Nn5g28145*) and *NnFLS* (*Nn5g30144*) was detected in petals transiently overexpressing *NnMYB5*. [Colour figure can be viewed at wileyonlinelibrary.com]

*MYB* transcription factors play a vital role in the regulation of anthocyanin and carotenoid biosynthesis. We identified 64 flavonoid‐related *MYB* genes in both American and Asian lotus (Table S17). Our phylogenetic tree revealed gene expansion in the TT2‐type MYB clade in *A. thaliana*, *N. nucifera* and *V. vinifera*, but not in *N. lutea* (Figure S8). For instance, five *MYB* genes are present on chromosome 4 in Asian lotus, but only two TT2‐type MYB orthologs exist in the American lotus genome (Figure 3a). Collinearity analyses indicated that the five *MYB* genes mentioned above were tandemly duplicated, forming an *MYB* gene cluster on chromosome 4 in the Asian lotus genome (Figure 3b). Further, repetitive DNA sequence contents in this *MYB* gene cluster genomic region are relatively higher in American lotus (15.39%) than in Asian lotus (13.20%). Two SVs observed in this region are likely to have resulted from variation in repetitive sequences between American and Asian lotus. The first SV resulted in a repeat contraction in American lotus, and the second SV resulted in a repeat contraction in American lotus (Figure 3b). Among the five Asian lotus *MYB* genes, only *Nn4g24223* (designated as *NnMYB5*) exhibited high transcript expression during petal development in Asian lotus. Additionally, transient overexpression of *NnMYB5* could induce anthocyanin accumulation in white petals of the Asian lotus cultivar (Figure 3c,d). The transcript abundances of *NnANS*, *NnCHS*, *NnFLS* and *NnF3H* were higher in *NnMYB5* transgenic petals, and therefore we predicted that those genes might be the targets of *NnMYB5* (Figure 3e).

### Thermogenesis and floral development

**Figure 4:**
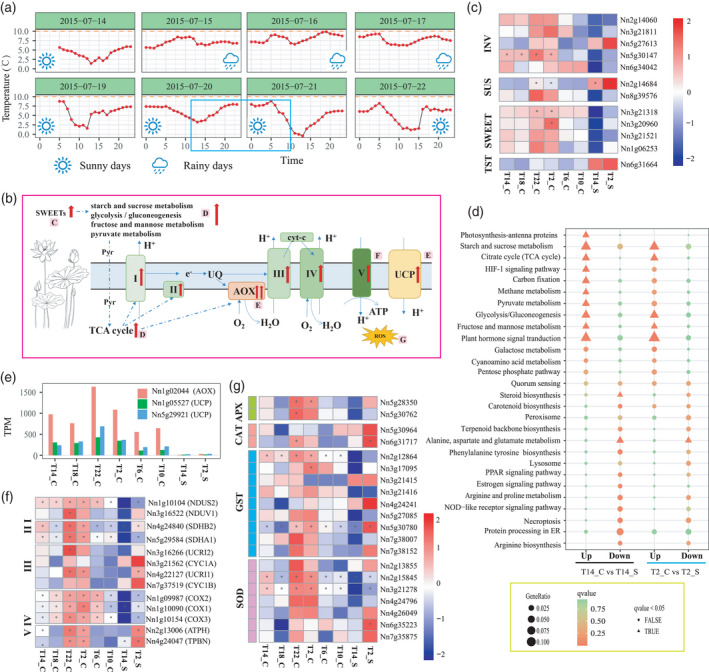
Floral thermogenesis in lotus. (a) Temperature differences (ΔT = T_lotus_ − T_air_) between the flower chamber of the lotus and the surrounding air over four consecutive days and nights. The blue box indicates the sampling period. (b) Schematic diagram of genes and processes involved in lotus thermogenesis. Abbreviations: I, NADH dehydrogenase; II, succinate dehydrogenase; III, cytochrome *c* reductase; IV, cytochrome *c* oxidase; V, ATP synthase; AOX, alternative oxidase; cyt‐c, cytochrome *c*; e^–^, electron flux; Glu, glutamate; Pyr, pyruvate; UCP, plant uncoupling protein; UQ, ubiquinone pool. (c) Heat map of differentially expressed genes (DEGs) encoding INVs (sucrose invertases), SUSs (sucrose synthases), SWEETs (sugar transporters known as ‘sugars will eventually be exported transporters’) and TSTs (tonoplastic sugar transporters) between carpels and stamens. (d) Comparison of KEGG enrichment for DEGs between carpels and stamens at 14:00 h and 2:00 h. (e) Transcript expression of the most abundant AOX and UCP candidates across all samples. (f) Heat map of the transcript expression of candidate genes related to the cytochrome pathway across all samples. (g) Heat map of the transcript expression of genes encoding enzymes related to antioxidant responses. [Colour figure can be viewed at wileyonlinelibrary.com]

Two sets of temperature data over four consecutive days and nights were collected during rainy and sunny days from 2 days before flowers opened. The temperature differences between the flower and the ambient air (ΔT) varied in different patterns. The highest ΔT was approximately 10°C at 18:00 h on a rainy day, and the lowest ΔT was approximately 0.2°C at 13:00 h on a sunny day. During the sampling period, ΔT increased at 22:00 h and remained high until 6:00 h the next morning (Figure 4a). By comparing the gene expression profiles of carpels with those of stamens, we found that differentially expressed genes (DEGs) with higher transcript abundances in carpels were enriched in pathways including ‘starch and sucrose metabolism’, ‘TCA cycle’, ‘glycolysis/gluconeogenesis’, ‘fructose and mannose metabolism’ and ‘plant hormone signal transduction’, both during the day and at night (14:00 h and 2:00 h). Whereas DEGs with higher transcript abundances in the stamens were enriched for ‘steroid biosynthesis’ only at 14:00 h (Figure 4d). Among the DEGs, we identified five sucrose invertase‐encoding (*INV*) genes, two sucrose synthase‐encoding (*SUS*) genes and four ‘sugars will eventually be exported transporters’‐encoding (*SWEET*) genes. Except for sucrose invertase *Nn5g27613* and sucrose synthase *Nn2g14684*, transcript abundances of the other genes, including *NnINV*s, *NnSUS*s and *NnSWEET*s, were significantly higher in carpels, especially at 22:00 h and 2:00 h (Figure 4c), suggesting possible active sugar transport and metabolism in carpels during this period. We identified two tonoplastic sugar transporters (*NnTST*s; *Nn5g31027* and *Nn6g31664*), but the transcript abundance of only *Nn6g31664* was significantly higher in stamens than in carpels (Figure 4c). Significant alterations in *AOX* transcript abundances in carpels were found during the sampled stages. The most abundant *AOX* gene, *Nn1g02044*, was the best match to *NnAOX1a* and *NnAOX1b*, which were reported to be responsible for thermogenesis in lotus (Grant et al., 2009), and was consistently more highly expressed in carpels than in stamens. At 22:00 h, when ΔT had increased, the transcript abundance of *Nn1g02044* was highest at 50–100‐fold than in stamens, but was significantly decreased by 6:00 h when the flowers first open (Figures 4e and S9). Six *NnUCP* orthologs were identified and the proteins encoded by the two highly expressed paralogs *NnUCP4* and *NnUCP5* (*Nn1g05527* and *Nn5g29921*) clustered phylogenetically with *AtPUMP5*, which functions in heat production (Figure S10). These two *UCP*s show similar expression profiles to those of *AOX* in carpels, stamens and floral receptacles during different thermogenic stages (Figures 4e and S9). In addition, orthologous genes related to the cytochrome pathway and antioxidant responses were co‐expressed with *AOX* (Figure 4f,g). In receptacles, the transcript abundances of most of the above‐mentioned genes also showed a strong correlation with thermogenic stages (Figure S9).

**Figure 5:**
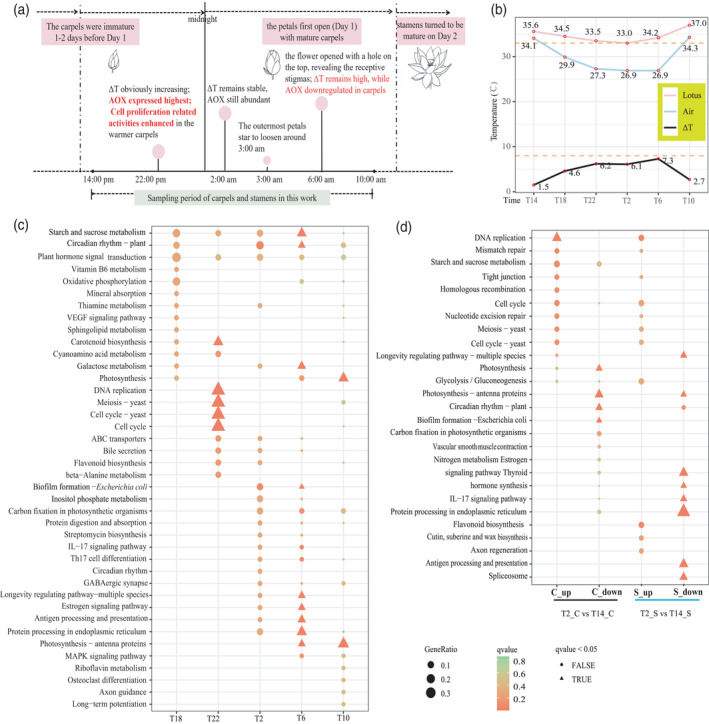
Thermogenesis and floral development. (a) Diagram showing the relationships between thermogenesis and gene expression profiles during lotus floral development; (b) Temperatures of lotus flower chamber and air at each sampling time. T14, T18, T22, T2, T6, and T10 represent 14:00 h, 18:00 h and 22:00 h on 21 July 2015 and 2:00 h, 6:00 h and 10:00 h on 22 July 2015, respectively. (c) KEGG enrichment of differentially expressed genes (DEGs) with increased transcript expression compared with earlier time point in the carpels. (d) Comparison of KEGG enrichment of DEGs at 2:00 h compared with those at 14:00 h in carpels and stamens. [Colour figure can be viewed at wileyonlinelibrary.com]

The maximal numbers of DEGs with increased or decreased transcript abundance occurred in comparisons of T10_C with T6_C and T2_S with T14_S (Figure S11), and our Kyoto Encyclopedia of Genes and Genomes (KEGG) analysis showed that these DEGs were enriched for photosynthesis‐related processes (Figure 5c,d). The temporal changes in DEGs in carpels and stamens, comparing 2:00 h with 14:00 h, varied for some biological processes (Figure 5d). At night, the transcript abundances of genes annotated for photosynthesis‐related processes decreased in both carpels and stamens. The transcript abundances of genes annotated for biological processes such as ‘protein processing in endoplasmic reticulum’ and ‘spliceosome’ were lower in the stamens, whereas those annotated for ‘DNA replication’‐related pathways were higher in the carpels (Figure 5d). Comparison of transcript abundances in carpels with previous time points showed that the transcript abundances of DEGs enriched in ‘DNA replication’ and ‘cell cycle’ were higher at 22:00 h, when ΔT substantially increases. The transcript abundances of DEGs enriched in ‘photosynthesis’ and ‘circadian rhythm’ continuously decreased from 14:00 h to 2:00 h, and then increased from 6:00 h to 10:00 h (Figures 4c and S11).

### Analyses of population structure, diversity and selective sweep

**Figure 6:**
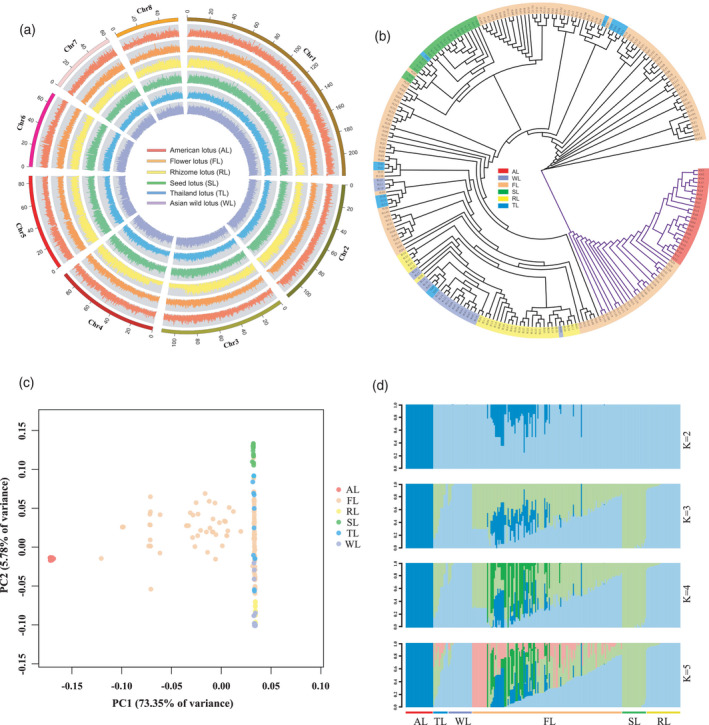
Population genetics of 240 lotus accessions. (a) The density distribution of single‐nucleotide polymorphisms (SNPs) for six lotus subgroups. The outermost ring indicates the chromosome (Chr), and the six inner rings represent the SNP density distribution of the six subgroups, including 24 American lotus (AL), 131 flowering lotus (FL), 30 rhizome lotus (RL), 21 seed lotus (SL), 13 Thailand lotus (TL) and 21 wild Asian lotus (WL). (b) Phylogenetic tree of 240 lotus accessions constructed from SNPs identified in the whole genome. (c) Principal component analysis (PCA) plot of 240 lotus accessions showing the first and second principal components. (d) Genetic structure of lotus. The number of assumed genetic clusters *K* ranged from 2 to 5. [Colour figure can be viewed at wileyonlinelibrary.com]

To explore genomic variation and diversity, we resequenced the genomes of 240 diverse lotus accessions worldwide, including 24 American lotus (AL) accessions and 216 Asian lotus accessions, which consisted of 13 Thailand lotus (TL), 21 wild lotus (WL), 131 flowering lotus (FL) cultivars, 21 seed lotus (SL) cultivars and 30 rhizome lotus (RL) cultivars (Figure S12). With an average mapping coverage of 10.84× for the Asian lotus genome, we identified 15 402 514 high‐quality single‐nucleotide polymorphisms (SNPs), 61.81% (58.28–65.03%) of which are located in intergenic regions (Figures 6 and S13; Table S18). A total of 7 174 284 common SNPs were observed among all subgroups. RL and FL accessions exhibited the highest (1 157 631, 8.20%) and lowest (385 539, 2.45%) percentages of unique SNPs, respectively (Figure S14; Table S19).

Phylogenetic tree and principal component analysis (PCA) were conducted using SNP variants detected in whole genomes (Figure 6b,c). These 240 accessions could be divided into two groups: AL accessions clustered together with 29 accessions that originated from interspecific hybridization with FL accessions, and another cluster consisting of Asian lotus accessions. The WL and RL accessions shared a closer phylogenetic relationship, whereas SL and TL accessions clustered together with the FL accessions. Structure analysis indicated that the deepest splits (*K* = 2) had occurred between American and Asian lotus (Figure 6d). Further splits (*K* = 3) occurred between the SL and WL plus RL accessions. Intriguingly, FL and TL accessions further diverged with increased *K*, and some FL accessions showed admixture with all of the ancestral genetic components of lotus, revealing their complex genetic backgrounds.

To investigate population divergence, we calculated the genetic diversity (*p*_*i*_ values) of each lotus subgroup and fixation statistics (*F*_ST_) between subgroups. The AL accessions showed higher nucleotide diversity (*p*_*i*_ = 1.38 × 10^−3^) than did the WL accessions (*p*_i_ = 0.58 × 10^−3^). Among the Asian lotus, FL accessions showed high nucleotide diversity (*p*_i_ = 4.23 × 10^−3^), followed by SL (*p*_i_ = 0.75 × 10^−3^) and RL (*p*_i_ = 0.63 × 10^−3^) accessions (Figure S15; Table S20). The high nucleotide diversity of the FL accessions was consistent with their highest heterozygosity (*Het* = 0.439%) among all the subgroups (Figure S13; Table S21). *F*_ST_ values revealed the highest degree of divergence between the AL and WL accessions (*F*_ST_ = 0.91) (Figure S15). In Asian lotus, high divergence was detected between the RL and SL accessions (*F*_ST_ = 0.676), and the next highest divergence was detected between the WL and SL accessions (*F*_ST_ = 0.669), whereas a low degree of divergence was detected between the SL and FL accessions (*F*_ST_ = 0.137). Moreover, the FL and TL accessions showed the least population divergence (*F*_ST_ = 0.04) (Table S20).

The *F*_ST_ estimates and the comparison of genetic diversity (*p*_i_ WL/*p*_*i*_ cultivated lotus *p_i_*_WL_/*p*_i__cultivated lotus_) were combined to identify selective sweeps during lotus domestication. We identified 206 SL‐selective sweeps between WL and SL, containing 412 genes, and 142 RL‐selective sweeps between WL and RL, containing 321 genes (Figure S16; Table S22). Notably, no selective sweep was identified between WL and FL. Only three sweeps and four genes overlapped for regions with selective signatures between SL and RL, suggesting that these three cultivated lotus groups were domesticated independently for particular traits and uses. Functional enrichment analysis showed that the genes that underwent selection in SL and RL were categorized in multiple metabolic pathways, such as cysteine and methionine metabolism in SL, and biosynthesis of secondary metabolites in RL (Figure S16b).

## DISCUSSION

American and Asian lotus share a series of morphological, physiological and genetic similarities, and have no reproductive barriers, although their offspring show low fertility (Huang, 1987). American lotus has been proposed as a subspecies of Asian lotus (Borsch & Ba Rthlott, 1994). However, several studies using molecular markers and genome resequencing analyses have reported that these two species show substantial genetic differentiation (Huang et al., 2018; Liu et al., 2020c). Thus, further comparison of the genomic diversity and variants between American and Asian lotus are necessary to resolve this issue. The 813‐Mb and 783‐Mb genomes of the Asian lotus varieties ‘China Antique’ and ‘Chinese Tai‐zi’, respectively, had been assembled and anchored into eight pseudochromosomes (Gui et al., 2018; Ming et al., 2013; Shi et al., 2020; Wang et al., 2013). Based on sequence data for the American lotus genome assembled here (843 Mb), the genome of American lotus is 30 Mb larger than that of Asian lotus ‘China Antique’, as a result of the higher number of repetitive DNA sequences in the American lotus genome (Figure 1a). Direct comparison of the genomes of American and Asian lotus indicates both high collinearity and extensive structural variation between these species (Figure 1c). In the two lotus species, 19.42% (6093) of genes contained SVs, which is lower than the 23.35% of such genes found between the *Zea mays* L. (maize) inbreds B73 and Mo17, and higher than the 15.29% of such genes found between the rice ‘Nipponbare’ and ‘R498’ genomes (Sun et al., 2018). The genomic variations currently apparent between American and Asian lotus were preceded by a long period of geographic isolation. Our phylogenetic tree and principal component analysis (PCA) suggested American lotus as the outgroup of Asian lotus (Figures 6 and S16). Population analysis also revealed the highest level of genomic divergence between AL and WL accessions (Figure S15). Combining the analyses of genomic and genetic divergence between American and Asian lotus, we reconfirmed the previous systematic designation of American and Asian lotus as two separate species of *Nelumbo*.

Flower color is a vital ornamental characteristic of lotus. We confirmed the presence of five anthocyanins in the flower petals of the Asian lotus ‘Chinese Antique’ but detected no anthocyanins in the petals of American lotus (Figure 2a). Aside from anthocyanins, other flavonoids accumulated to higher levels in American lotus than in Asian lotus (Figure S7). We qualitatively and quantitatively analyzed the content of carotenoids for the first time, to our knowledge, in American lotus and identified the carotenoid compounds that could contribute to the yellow flower color in this lotus species. The yellow color in petals of American lotus arise through the accumulation of lutein, β‐carotene, neoxanthin and other carotenoids (Figures 2b and S7). The early genes in the anthocyanin biosynthesis pathway and structural genes in the carotenoid biosynthesis pathway displayed higher expression in American lotus petals than in Asian lotus petals, with only the late genes in the anthocyanin biosynthesis pathway exhibiting higher transcript expression in Asian lotus than American lotus petals (Figure 2c,d). The structural genes of the anthocyanin and carotenoid biosynthesis pathways are conserved between American and Asian lotus, and SVs between the two species were detected in the exons of three genes, including *F3′H*, *ANGT* and *PSY* (Table S16). As the *ANGT* gene exhibited little or no transcript expression during pigment accumulation in petals, it unlikely to be the key gene resulting in the flower color difference between American and Asian lotus. *PSY* encodes the enzyme that performs the rate‐limiting step in carotenoid biosynthesis and is transcriptionally regulated by hormones and external environmental stimuli (Lindgren et al., 2003). The *PSY* gene exhibits high transcript expression in flower petals of American lotus, but relatively low expression in red petals of Asian lotus, resulting in lower carotenoid content in Asian lotus (Figure 2d). Genes encoding regulatory transcription factors, such as *MYB*s, influence pigment accumulation by regulating the expression of pigment biosynthesis genes. For example, *R2R3*‐*MYB* transcription factors regulate both anthocyanin and carotenoid biosynthesis (Chiu et al., 2010; Quattrocchio et al., 1999; Sagawa et al., 2016). We detected SVs in the genomic region containing a cluster of putative anthocyanin‐regulatory *MYB* genes on chromosome 4 in lotus. Five duplicated *MYB* genes were detected in this cluster in the Asian lotus genome, but only two *MYB*‐encoding genes were found in the corresponding genomic region of American lotus (Figure 3b). The higher repetitive sequence content of the American lotus genome comprises predominantly *Gypsy* long terminal repeats (LTRs), and two large SVs in a collinear region between the American and Asian lotus genomes are likely to have resulted from repetitive expansion and contraction in this region. We propose that transposon activity might have played a role in structural variation in this region. The *NnMYB5* sequence from the *MYB* cluster was used to test its putative function in anthocyanin biosynthesis in lotus petals. Because transient overexpression of *NnMYB5* in white petals of Asian lotus could induce anthocyanin accumulation (Figure 3), we have designated *NnMYB5* as the candidate gene that could positively regulate anthocyanin biosynthesis in Asian lotus by activating the expression of anthocyanin biosynthesis genes, such as *NnANS*, *NnCHS* and *NnFLS*, but fails to activate the expression of anthocyanin biosynthesis genes in American lotus because of the absence or inadequate expression of its transcripts.

The phenomenon of floral thermogenesis has been discovered in a diverse range of plant taxa. Consistent with previous observations, lotus flowers are able to regulate their internal temperature to keep them warm during low ambient temperatures at the floral receptivity stage (Figure 4a). The floral receptacle of lotus is the main source of heat during the thermogenic stage (Grant et al., 2008; Miller et al., 2009; Watling et al., 2006). Although lotus carpels are enclosed in the warm spongy tissue of the thermogenic receptacle, it has not been clear whether carpels could produce heat independently. Evidence indicates that AOX is responsible for controlling thermogenesis in lotus (Sun et al., 2021; Watling et al., 2006), which is mainly fueled by carbohydrates (Grant et al., 2008). We found that the candidate gene for *AOX* with the highest transcript abundance, *Nn1g02044*, was consistently more highly expressed in carpels than in stamens, where its transcript abundance can reach 50–100‐fold of that found in stamens (Figure 4e). High fluxes of AOX are required for achieving significant heat production, whereas low rates of AOX flux in non‐thermogenic tissues or stages cannot generate measurable heat (Breidenbach et al., 1997). Transcript abundances of genes involved in biological processes, including ‘starch and sucrose metabolism’, ‘TCA cycle’, ‘glycolysis/gluconeogenesis’, ‘fructose and mannose metabolism’ and ‘pyruvate metabolism’, were also enhanced in thermogenic carpels (Figure 4d). In most plants, sucrose is the end product of photosynthesis that is transported from source to sink tissues and the entry of carbon from sucrose into cellular metabolism can potentially be catalyzed by either INVs or SUSs (Barratt et al., 2009). Compared with stamens, the transcript abundances of most of the differentially expressed *INV* and *SUS* genes increased in a temperature‐correlated manner in carpels (Figure 4c). Sugar transporters such as SWEET mediate sucrose efflux, a key step for phloem transport, and are major players in governing the long‐distance distribution of sugars throughout the plant (Chen et al., 2012). The transcript abundances of all differentially expressed *SWEET* genes were also increased in carpels (Figure 4c). Thus, in addition to enhanced sugar metabolism and transportation, high fluxes through AOX could allow carpels to produce heat independently, with sugars used as the main substrates. In addition to the AOX pathway, another energy‐dissipating system mediated by UCPs is also involved in plant thermogenesis. We found that the two UCP‐encoding genes with the highest transcript abundance were closely phylogenetically clustered with AtPUMP5, and showed expression profiles similar to that of *AOX* across all samples as well as in receptacles during different thermogenic stages (Figures 4e and S9). Similarly to AtPUMP1, AtPUMP5 can induce a linoleic acid‐mediated H^+^ flux that is sensitive to ATP and GTP (Borecky et al., 2006). Our phylogenetic and expression analyses suggest that UCPs might also contribute to thermogenesis in lotus, in addition to AOX. Under such high demand for heat generation, both pathways might work together to meet demand. In addition, cytochrome pathway‐related candidate genes show expression patterns similar to that of *AOX*, indicating the presence of the intensive cellular respiratory activity that is likely to occur during carpel thermogenesis (Figure 4e). Meanwhile, transcript abundances of most of the genes encoding enzymatic antioxidants, such as ascorbate peroxidase (APX), catalase (CAT), glutathione‐*S*‐transferase (GST) and superoxide dismutase (SOD), showed expression profiles similar to that of *AOX*, indicating that an antioxidant response system and reactive oxygen species (ROS) signaling might be induced during thermogenesis (Figure 4g). As AOX can dissipate excess redox potential and UCP can dissipate the proton motive force, the simultaneous operation of these two energy‐dissipating systems could also play important roles in stress responses (Arnholdt‐Schmitt et al., 2006; Barreto et al., 2020). As shown above, transcript abundances of genes involved in cellular respiratory activities were considerably stimulated and those of genes involved in antioxidant response systems were induced during thermogenesis. Thus, the co‐expression of AOX and UCPs might also play roles in the maintenance of redox homeostasis under such intensive metabolic conditions (Figure 4b).

Thermogenesis is a very energy‐intensive process and should be important to plant reproduction and survival. Previous studies on the function of thermogenesis in lotus have mainly focused on pollination biology. Here, we found that the function of thermogenesis in lotus seems to be closely related with protogyny (Figure 5a). During the sampling stages, ΔT increased from 22:00 h and remained high until 6:00 h (Figure 5b). However, the transcript abundances of AOX and other related genes were highest at 22:00 h, then significantly decreased at 6:00 h (Figure 4). As high fluxes of AOX are required for achieving significant heat production, this suggests that thermogenesis in carpels decreased or stopped by 6:00 am. Then a question was raised regarding what caused this shift in thermogenic activity in carpels in the morning. We found that the transcript abundance of DEGs enriched in putative ‘DNA replication’ and ‘cell cycle’ functions increased from 22:00 h in carpels (Figure 5c). Comparisons of DEGs between carpels and stamens at 2:00 h and 14:00 h revealed that most DEGs with decreased transcript abundance were enriched in ‘photosynthesis’, whereas DEGs enriched in ‘DNA replication’ increased specifically in carpels (Figure 5d). These results suggested that cell proliferation‐related activities might be specifically enhanced or maintained in the warmer carpels when the ambient temperature decreased. Lotus carpels are immature 1–2 days before the petals first open (day 1) and become mature on day 1 (Seymour & Schultze‐Motel, 1998). According to previous observations on lotus anthesis under similar conditions, the outermost petals start to loosen around 3:00 h, and the flowers open partially at around 5:30 h to reveal the receptive stigmas on the morning of day 1 (Huang, 1982; Seymour & Schultze‐Motel, 1998). Most of the carpels are then fertilized with pollen from other plants with the aid of insect pollinators. Subsequently, the stamens mature and the flowers open widely the next day (day 2) (Seymour & Schultze‐Motel, 1998). Combined with those previous observations of lotus flowering (Guozhen, 1982; Seymour & Schultze‐Motel, 1998), the increases observed in transcript abundances of DEGs with cell proliferation‐related activities were specifically found in warmer carpels during the cold night before the first blooming (Figure 5c,d), and suggest that thermogenesis might promote carpel development and be related to protogyny in lotus. The temperature difference between the carpels and stamens might promote the temporal separation of sexual maturity to prevent self‐pollination.

Transcripts of *INV*s, *SUS*s and *SWEET*s were highly expressed during thermogenesis, but the *TST*s that move sucrose into and out of vacuoles were not more highly expressed during thermogenesis, indicating that the sugars used as substrates for thermogenesis could have come from source leaves via long‐distance sugar transporters, encoded by *SWEET*s. The UCP pathway, in addition to AOX, also contributes to thermogenesis, and we found that *CUP4* and *CUP5* were highly expressed during thermogenesis. The involvement of their orthologous genes in heat production in Arabidopsis has also been shown (Borecky et al., 2006).

Asian lotus has been domesticated for specialized flower, seed or rhizome production. *F*_ST_ analysis indicated that accessions of seed lotus and rhizome lotus have experienced a large degree of population divergence, whereas accessions of seed lotus and flowering lotus have experienced a low level of divergence (Figure S13). The divergence among accessions of flowering lotus, seed lotus and rhizome lotus is likely to have arisen from the preferred targeting of different traits for improvement during domestication (Yang et al., 2015). Moreover, flowering lotus accessions show higher nucleotide diversity and the highest heterozygosity (Table S21), which can be explained by their complicated origin during artificial selection (Liu et al., 2020c). Notably, the wild lotus accessions showed lower genetic diversity and heterozygous ratio than the cultivated lotus accessions (Tables S20 and S21), which might be because of their isolated habitat and asexual reproduction by rhizomes as the main reproduction mode. Wild lotus germplasm resources can be used in research on the origin and breeding of lotus. However, wild lotus accessions suffer several threats, from environmental pollution, habitat loss and the undesirable admixture of these wild populations with domesticated accessions (Liu et al., 2020c). Therefore, it is urgent to strengthen the protection of wild lotus resources. As a result of higher genetic diversity and complex introgression from wild accessions and interspecific hybridizations, no selective sweeps were identified in flowering lotus. However, specific selective sweeps were detected in seed lotus and rhizome lotus (Figure S14), which suggests that these three cultivated lotus groups were domesticated independently for particular traits and uses. The genes underlying the selective sweeps in seed lotus and rhizome lotus will provide genomic resources for the molecular breeding of lotus.

In summary, our study presents a high‐quality chromosome‐scale genome assembly of American lotus that will be useful for comparing the genetic divergence of and variants between American lotus and Asian lotus. Comparative genomic analyses showed the two species exhibited high synteny and diverged about 13.86 Mya. A total of 29 533 SVs were detected genome‐wide between American lotus and Asian lotus, including two large variants in the *MYB* gene cluster region. Although transient overexpression experiments confirmed that *NnMYB5* could induce anthocyanin synthesis to produce red coloration in white Asian lotus petals, this gene is not expressed in American lotus; therefore, *NnMYB5* was designated as a candidate gene for the control of differentiation in flower petal color between American lotus and Asian lotus. Increased transcript abundances of genes encoding AOX, UCPs and sugar metabolic enzymes and transporters suggest that these genes are likely to contribute to thermogenesis, which might promote carpel development and affect reproduction in lotus. The population genomic analyses in the present study revealed deep divergence between American and Asian lotus, and the independent domestication of seed, rhizome and flower traits in cultivated lotus. These results revealed possible genomic bases and identified candidate genes possibly involved in petal color, thermogenesis and domestication in lotus.

## EXPERIMENTAL PROCEDURES

### Plant materials

To explore the genome of American lotus, *N. lutea*, we sampled fresh leaves of American lotus ‘AL1’ to isolate genomic DNA for PacBio sequencing and Illumina HiSeq sequencing, and chromatin for Hi‐C sequencing. To annotate genes and identify species‐specific genes, leaf, petiole, flower, root and rhizome tissues from ‘AL1’ and from Asian lotus ‘China Antique’ were mixed for isoform sequencing (ISO‐seq). Asian lotus cultivar ‘Jianxuan 17’ was selected for RNA‐seq analysis of thermogenesis. To isolate genomic DNA for genome resequencing, we picked fresh leaves of 240 lotus accessions, including 216 Asian lotus accessions and 24 American lotus (AL) accessions (Figure S13). These Asian lotus accessions included 13 Thailand lotus (TL), 21 wild lotus (WL), 131 flowering lotus (FL) cultivars, 21 seed lotus (SL) cultivars and 30 rhizome lotus (RL) cultivars. All materials were obtained from the Wuhan Botanical Garden, Chinese Academy of Sciences.

### Genome assembly

The genome of American lotus, *N. lutea*, was assembled by incorporating PacBio long reads and Illumina short reads as well as Hi‐C technology. A total of 74.6 Gb of long reads generated using the PacBio RSII platform were self‐corrected, trimmed and assembled using the canu 1.9 assembler (Koren et al., 2017), with default parameters. pilon (Walker et al., 2014) was used to correct base calls and InDels inaccurately assembled in the genome. We aligned the raw PacBio reads against the initial assembly using minimap2 (Li, 2018) and calculated the read depth for each contig. The alternative contigs were removed from the initial assembly using purge_haplotigs (Roach et al., 2018). The resulting contigs were further subjected to Hi‐C scaffolding using the allhic pipeline (Zhang et al., 2019). The embryophyta_odb10 database, which includes a total of 1375 ultra‐conserved plant proteins, was used for the assessment of genome assembly and annotation.

### Genome annotation

The annotation of the American lotus genome included the annotation of transposable elements and RNA genes. To annotate repeat sequences in the genome, repeatmodeler (https://github.com/Dfam‐consortium/RepeatModeler) was initially applied to train the novel repeat library. repeatmodeler executed the repeat searching algorithms recon (Bao & Eddy, 2002) and repeatscout (Price et al., 2005). The customized repeat library was further used as a query to predict repetitive sequences in repeatmasker (https://github.com/rmhubley/RepeatMasker). We used geta (https://github.com/chenlianfu/geta), augustus (Stanke et al., 2006), Trimmomatic (Bolger et al., 2014), hisat2 (Pertea et al., 2016) and genewise (Birney et al., 2004; Birney & Durbin, 2000) to annotate RNAs. RNA‐seq data were first trimmed using trimmomatic and further aligned against the reference ‘AL’ genome using hisat2 (Pertea et al., 2016). The geta pipeline was used to predict reliable introns and optimize transcripts. Transcripts were then subjected to open reading frame (ORF) prediction in transdecoder (https://github.com/TransDecoder/TransDecoder). Based on their intron and exon structures, gene models were then iteratively trained using augustus until the best score was obtained. Homologous proteins, including those from *A. thaliana*, *O. sativa* and *N. nucifera*, as well as *Medicago truncatula*, *Phaseolus vulgaris* and *Solanum lycopersicum*, were downloaded from Phytozome 12 (https://phytozome.jgi.doe.gov/pz/portal.html) and subjected to genewise for further protein prediction. The Pfam database was used to screen for high‐quality gene models and a final gene prediction data set, including CDS, protein sequences and a GFF3 file locating gene positions, was released based on the aforementioned evidence using a perl script (combinegenemodeler) implemented in the geta pipeline. Gene annotation was further assessed using BUSCO (Simao et al., 2015), with embryophyta_odb10 as the testing database.

### Phylogenetic reconstruction and divergence time estimation

orthomcl (Li et al., 2003) was used to identify homologs and single‐copy orthologous genes in *A. thaliana*, *C. papaya*, *M. integrifolia*, *N. lutea*, *N. nucifera*, *O. sativa*, *S. lycopersium* and *V. vinifera*. The maximum‐likelihood phylogenetic tree was constructed by raxml (Alexandros and Stamatakis, 2014) with an alignment file of single‐copy protein sequences from the above seven species. mcmctree in paml (Yang, 2007) was used for estimating the divergence times. Two divergence times, 68–72 Mya, between *A. thaliana* and *C. papaya*, and 120–140 Mya, between monocot and eudicot, were used for calibration.

### Detection of structural variants between American and Asian lotus

nucmerin mummer (Kurtz et al., 2004) was used for all‐versus‐all comparison of the genome sequences of American lotus and Asian lotus (Marcais et al., 2018; Shi et al., 2020). The alignment output files were filtered using delta‐filter, unique alignment longer than 1000 bp and identity no less than 90, and were then used for downstream analysis. We further analyzed the SVs using the online program assemblytics(Maria & Schatz, 2016) with the parameters unique sequence length longer than 10 kbp and variant size range from 50 to 10 000 bp. GO enrichment analysis for the SV‐related genes was performed using the r package clusterprofilerwith the Benjamini‐Hochberg *P*‐value adjusted method with an adjusted *P*‐value cut‐off of <0.05.

### Species‐specific gene identification in American and Asian lotus

BLASTP was used to identify species‐specific genes in American and Asian lotus. Predicted proteins of American lotus with no hits in the protein set of Asian lotus were considered as American lotus species‐specific genes. Putative protein‐coding sequences of those potentially species‐specific genes were further confirmed by BLAST against the Asian lotus genome sequence and resulted in no hits, with an e‐value cut‐off of 1e‐6. Asian lotus species‐specific genes were identified in the same way.

### Determination of anthocyanins and carotenoids in the petals of American and Asian lotus

Petals from flowers of American lotus and Asian lotus were collected on the first day of flowering and frozen in liquid nitrogen. Flavonoids in the petals were extracted as reported by Deng et al. (2013). Standards of quercetin 3‐*O*‐galactoside, quercetin3‐*O*‐glucoside, isorhamnetin 3‐*O*‐rutinoside, kaempferol 3‐*O*‐glucoside and malvidin 3‐*O*‐glucoside were purchased from Shanghai Yuanye Bio‐technology Company. An Agilent 1290 HPLC system and a Sunfire C18 column (4.6 × 150 mm, 3.5 μm; Waters, https://www.waters.com) were used for chromatographic separation of pigments.

The carotenoids were isolated from the petals of American lotus and Asian lotus as reported previously, with some modifications, and the extracted supernatant was filtered on a 0.22‐μm membrane filter before use in further liquid chromatography with tandem mass spectrometry (LC‐MS/MS) analysis (Inbaraj et al., 2008; Liu et al., 2020b). Carotenoids were analyzed using a UPLC‐APCI‐MS/MS system (UPLC, ExionLC™ AD, Applied Biosystems 6500 Triple Quadrupole; Applied Biosystems, now ThermoFisher Scientific, https://www.thermofisher.com). The analytical conditions we used followed the methods in Geyer et al. (2004). Finally, carotenoid contents were detected using metware (http://www.metware.cn), based on the AB Sciex QTRAP 6500 LC‐MS/MS platform.

### Structural variants and expressions of genes related to anthocyanin and carotenoid biosynthesis in American and Asian lotus

Sequence of anthocyanin biosynthesis genes in Arabidopsis were downloaded from TAIR (https://www.arabidopsis.org) as queries to identify homologs in *Nelumbo* species by BLASTP with an e‐value of <1e‐6. The obtained genes were confirmed by BLAST against the NR database in NCBI (Table S16). Genes in the carotenoid biosynthesis pathway were identified using a similar method as above with homologous proteins from *Daucus carota* subsp. *sativus* (carrot) as queries (Ma et al., 2018). *MYB* genes in Arabidopsis and grape were downloaded from the Phytozome 12 database (https://phytozome.jgi.doe.gov/pz/portal.html). Phylogenetic trees were generated using the website at ATGC (http://www.atgc‐montpellier.fr/phyml/) (Guindon et al., 2010) and multiple sequence alignments were performed using clustalw. Collinearity analysis was conducted using mcscan (Tang et al., 2008). Genomic structural variants in, near or containing genes were queried in the genomic structural variants file by gene position.

Mixed tissues from leaves, petioles, flowers, roots and rhizomes of American lotus ‘AL1’ or Asian lotus ‘China Antique’ were sequenced using long‐read isoform sequencing. Sequencing libraries were prepared following the protocols of the SMARTer PCR cDNA Synthesis Kit (Clontech, now TaKaRa, https://www.takarabio.com) and the BluePippin Size Selection System (PacBio, https://www.pacb.com), and sequencing was performed on a PacBio RSII sequencing platform. Subreads were filtered and subjected to CCS using the SMRT Analysis Server 2.2.0 (PacBio). Gene counts were calculated using htseq‐count (Anders et al., 2015).

Petals at the developmental stages of S1 (about 2–3 cm) and S2 (about 5–6 cm) were collected from the American lotus ‘AL1’ and Asian lotus ‘China Antique’ flower buds for total RNA isolation. We performed quantitative reverse‐transcription polymerase chain reaction (qRT‐PCR) on a StepOnePlus™ Real‐Time PCR System (ThermoFisher Scientific, https://www.thermofisher.com) with TaKaRa SYBR^®^ Premix Ex Taq™ (TaKaRa, https://www.takarabio.com) Conserved regions of homologs between two species were used for primer design using prime primer 3 (https://primer3plus.com). An *actin* sequence from Asian lotus (*Nn7g36007*) was used as the internal reference, and the 2^–Δ*C*t^ method was used to calculate relative expression. The pairs of gene‐specific primers used for qRT‐PCR are listed in Table S23.

### Transient expression of *NnMYB5* genes in lotus petal

The coding region of *NnMYB5* was cloned from cDNA of ‘China Antique’ lotus and cloned into the pMDC43 vector using the Gateway protocol to construct the *35S*:*NnMYB5* overexpression plasmid. A single colony of *Agrobacterium tumefaciens* strain *GV3101* carrying the *35S*:*NnMYB5* plasmid was inoculated into LB liquid and grown overnight at 28°C, and then suspended in infiltration buffer (10 mmMgCl_2_, 10 mm2‐(*N*‐morpholine)‐ethanesulphonic acid (MES), 150 mmacetosyringone, pH 5.6) to OD_600_ = 0.2. The infiltration medium was then injected with a syringe into lotus petals at 3 days before flowering. *Agrobacterium* tumefaciens strain *GV3101* containing empty pMDC43 was used as a control. On the third day after injection, the contents of anthocyanin were analyzed and total RNA was extracted to detect the relative expression of genes by qRT‐PCR.

### RNA‐seqanalysis of thermogenesis

The temperatures of Asian lotus cultivar ‘Jianxuan 17’ flowers (carpels) and the ambient air were monitored and measured with an electronic thermometer every other hour over four consecutive days and nights, beginning at 5:00 h 2 days before the petals opened. Two sets of temperature data were collected during rainy and sunny days. The carpels and stamens were sampled from 14:00 h on 12 July 2015 to 10:00 h on 22 July 2015 with three biological replicates for RNA‐seq. Carpels were collected at six time points, including 14:00 h, 18:00 h, 22:00 h, 2:00 h, 6:00 h and 10:00 h, whereas stamens were only collected at 14:00 h and 2:00 h. RNA was extracted from approximately 150 mg of tissue with TRIzol reagent following the manufacturer’s protocol. The Agilent 2100 BioAnalyzer (Agilent, https://www.agilent.com) was used to determine the quality and integrity of each sample RNA. RNA‐seq libraries were constructed separately using the NEB Next Ultra RNA Library Prep Kit for Illumina (New England Biolabs, https://international.neb.com), according to the manufacturer’s protocol. Each library was evaluated by electrophoresis on 0.8% agarose and quantified using a fluorometer. A total of 24 cDNA libraries were sequenced on an Illumina platform (HiSeq2500; https://emea.illumina.com) to generate a larger number of 150‐nt paired‐end sequence reads. The sequence data were quality checked using fastqc and trimmed using fastp (Andrews, 2014; Chen et al., 2018). About 4 Gb of clean data were generated and then mapped onto the Asian lotus reference genome (‘China Antique’ v2.0, http://nelumbo.biocloud.net) using hisat2 with default parameters. The count matrix and transcripts per million (TPM) matrix corresponding to each gene were generated using featurecounts (Kim et al., 2019; Liao et al., 2014; Pertea et al., 2015). Subsequently, the count matrix was imported into the deseq2 package for the identification of DEGs (Love et al., 2014). Genes with a false discovery rate (FDR) lower than 0.05 were considered significantly differentially expressed between different groups. GO and KEGG enrichment analysis were performed using the r package clusterprofiler (Yu et al., 2012).

For a more comprehensive understanding of the mechanism of lotus thermogenesis, published RNA‐seq data for receptacles during five thermogenic stages were downloaded from the NCBI Sequence Read Archive (SRA) with the accession number PRJNA548651 (Liu et al., 2020a). First, reads were transformed into fastq files using the SRA toolkit. Then the fastq files were processed using similar method as above and a TPM count matrix was generated for downstream analysis. The protein sequences of AOX, UCP, cytochrome pathway I–V genes, enzymatic antioxidant, SUS, INV and SWEET genes were downloaded from the Swiss‐Prot database (https://www.uniprot.org) and used as queries to identify homologs in the Asian lotus ‘China Antique’ reference genome using BLASTP with an e‐value cut‐off of <1e‐10. TST protein sequences from Arabidopsis were used as queries to identify homologs in Asian lotus by BLASTP with an e‐value cutoff of <1e‐100. Candidate genes were retained after filtering out genes with low expression across all samples (max. TPM < 10). Then, candidate SWEETs with the MtN3_slv domain, candidate SUSs with the SUS domain and glycosyl transferase group 1 domain, and candidate INVs with Glyco_hydro_100, Glyco_hydro_32C or Glyco_hydro_32N domains were retained for further analysis. Heat maps were generated using the pheatmap package in r  4.1.1. Bar charts and line charts were generated using r  4.1.1. With reference to Hourton‐Cabassa et al. (2004), amino acid sequences of UCP from Arabidopsis, rice, other plants and other organisms, as well as six lotus UCP candidates, were used for phylogenetic analysis. The best amino acid substitution models were calculated using prottest  3.4 (Darriba et al., 2011). A maximum‐likelihood (ML) tree was then reconstructed using raxml  8.2.11 (Stamatakis, 2014) with the PROTGAMMAJTTF model and 1000 bootstrap replications.

### Genome resequencing, SNPcalling, and population structure in American and Asian lotus

A total of 240 lotus accessions were chosen for genome resequencing and population structure analyses. Sequencing libraries were generated using the TruSeq Nano DNA HT Sample Preparation Kit (Illumina), following the manufacturer’s instructions and were sequenced on the Illumina HiSeq PE150 platform. Low‐quality paired reads were filtered out of the data. The clean reads were mapped to the Asian lotus ‘China Antique’ reference genome using the burrows‐wheeler aligner (bwa) and sorted bam files were obtained using samtools 1.9 (Li & Durbin, 2009; Shi et al., 2020). sentieon (http://www.sentieon.com) was used to identify the raw SNPs, and only high‐quality SNPs (coverage depth ≥ 4, RMS mapping quality ≥ 20, maf ≥ 0.05, miss ≤ 0.1) were retained for subsequent analysis. SNP annotation was performed using annovar (Wang et al., 2010). The neighbor‐joining (NJ) method based on the p‐distance using treebest 1.9.2 was used to construct an individual‐based phylogenetic tree that was visualized using mega 6 (Tamura et al., 2013). gctawas used to carry out principal component analysis (PCA) (Yang et al., 2011). admixturewas used to perform an unsupervised cluster analysis, with the number of assumed genetic clusters *K* ranging from 2 to 5 (Alexander et al., 2009).

### Identification of selected regions of cultivated Asian lotus

A genome‐wide analysis of selective sweeps in cultivated Asian lotus was implemented according to previous studies (Li et al., 2013; Zhao et al., 2021). Here, we calculated the genome‐wide distribution of the *F*_ST_ values and *p*_*i*_ ratios for the defined group pairs (WL–SL and WL–RL). The *p*_*i*_ ratios (*p*_*i*__WL_/*p*_*i*__cultivated lotus_) were log_2_‐transformed. Subsequently, we estimated and ranked the empirical percentiles of *F*_ST_ and log_2_(*p*_*i*_ ratio) in each window, and the windows with the top 5% *F*_ST_ and log_2_(*p*_*i*_ ratio) values simultaneously were considered as regions exhibiting strong selective sweep signals. KEGG enrichment analysis was implemented using the KOBAS 3.0 server (Xie et al., 2011).

## CONFLICT OF INTEREST

The authors declare that they have no conflicts of interest associated with this work.

## AUTHOR CONTRIBUTIONS

MY and RM conceived this genome project and coordinated the research activities. MY and RM designed the experiments. MY collected Asian lotus germplasm and WOO collected American lotus germplasm. XZ, JL and XX assembled and annotated the genome. JL and HS analyzed petal color; PZ and YQ analyzed thermogenesis. HS, XD and DY analyzed re‐sequenced genomes. JL, WZ, MW and YZ analyzed RNA‐seq data. YH analyzed the American lotus karyotype. YQ, HS, JL, PZ, MY and RM wrote the article.

## Supporting information


**Figure S1.** Hi‐C mapping of eight chromosomes of American lotus.
**Figure S2.** Kimura distance analysis of transposable elements in the American lotus genome.
**Figure S3.** Shared gene families among *Arabidopsis thaliana* (At), *Carica papaya* (Cp), *Nelumbo lutea* (Nl), *Nelumbo nucifera* (Nn), *Oryza sativa* (Os), and *Vitis vinifera* (Vv).
**Figure S4.** Distribution of 67 genes specific to American lotus on eight American lotus chromosomes.
**Figure S5.** Size distribution of structure variants between American and Asian lotus detected using Assemblytics.
**Figure S6.** Size distribution of structure variants between American and Asian lotus detected using assemblytics.
**Figure S7.** Non‐anthocyanin flavonoids and xanthophylls in American and Asian lotus.
**Figure S8.** Phylogenetic tree of *MYB* genes involved in flavonoid biosynthesis in *Amborella trichopoda* (Am), *Arabidopsis thaliana* (At), *Nelumbo lutea* (Nl), *Nelumbo nucifera* (Nn), *Vitis vinifera* (Vv).
**Figure S9.** Thermogenesis in floral receptacles during different stages of flowering.
**Figure S10.** A phylogenetic tree based on the amino acid sequences of UCPs from plants including *Arabidopsis thaliana*, *Mangifera indica*, *Nelumbo nucifera*, *Oryza sativa*, *Phodopus sungorus*, *Solanum lycopersicum* and *Zea mays*, as well as other animal UCPs from *Bos taurus*, *Canis lupus familiaris*, *Cyprinus carpio*, *Danio rerio*, *Dicrostonyx groenlandicus*, *Drosophila melanogaster*, *Homo sapiens*, *Mus musculus*, *Mesocricetus auratus*, *Ochotona dauurica*, *Oryctolagus cuniculus*, *Ovis aries*, *Pongo abelii*, *Rattus norvegicus*, *Suncus murinus* and *Sus scrofa*.
**Figure S11.** DEGs in carpels identified by comparing each time point with the previous time point.
**Figure S12.** Geographic distributions and components of lotus accessions.
**Figure S13.** Sequencing depth and heterozygosity of 240 lotus accessions.
**Figure S14.** Venn diagram of SNP number between different lotus groups.
**Figure S15.** Population divergence and nucleotide diversity of different lotus subgroups.
**Figure S16.** Selection in cultivated Asian lotus.

**Table S1.** Sequencing information.
**Table S2.** Statistics of contig‐level genome assembly and annotation.
**Table S3.** Chromosome‐scale genome assembly.
**Table S4.** Assessment of genome assemblies based on assembled RNA‐seq transcripts.
**Table S5.** Annotation of transposable elements (TEs).
**Table S6.** BUSCO completeness analysis of genome assembly and annotation.
**Table S7.** Overall orthogroups in *Arabidopsis thaliana*, *Carica papaya*, *Oryza sativa*, *Nelumbo lutea*, *Nelumbo nucifera*, and *Vitis vinifera*.
**Table S8.** Statistics of orthogroups in *Arabidopsis thaliana*, *Carica papaya*, *Oryza sativa*, *Nelumbo lutea*, *Nelumbo nucifera*, and *Vitis vinifera*.
**Table S9.** List of genes specific to Asian lotus or American lotus.
**Table S10.** List of GO terms enriched in Asian lotus‐specific genes.
**Table S11.** Structural variant statistics.
**Table S12.** Genomic distribution of structural variants.
**Table S13.** List of GO terms enriched in genes affected by structure variants.
**Table S14.** Genes in the anthocyanin biosynthesis pathway of American and Asian lotus.
**Table S15.** Genes in the carotenoid biosynthesis pathway of American and Asian lotus.
**Table S16.** List of flavonoid and carotenoid biosynthesis genes with structural variation between American and Asian lotus.
**Table S17.** Genes used for phylogenetic analysis of flavonoid biosynthesis related to *MYB* genes.
**Table S18.** Summary of SNPs from all varieties of lotus.
**Table S19.** Statistics of common and unique SNPs across different subgroups.
**Table S20.** Statistics of *p*_*i*_ and *F*_ST_ across different subgroups.
**Table S21.** The heterozygosity ratio in each subgroup.
**Table S22.** Genes under selection associated with domestication in seed and rhizome lotus accessions.
**Table S23.** List of primers used for qRT‐PCR in this study.

## Data Availability

PacBio whole‐genome sequencing data, Illumina data, genome resequencing data and RNA‐seq data have been deposited in NCBI (https://www.ncbi.nlm.nih.gov/). The genome assemblies and gene annotations have been deposited under BioProject number PRJNA747731.The RNA‐seq data for thermogenesis have been deposited under BioProject number PRJNA748519. The raw data for resequencing genome of 240 lotus accessions have been deposited under BioProject number PRJNA749672.
