# Supplementary material for: Comparative analyses of American and Asian lotus genomes reveal insights into petal color, carpel thermogenesis and domestication
Source: Plant J. 2022 Apr 19;110(5):1498–515. doi: 10.1111/tpj.15753 (PMC9325450; doi:10.1111/tpj.15753)
Supplement: Supplementary file 1 — Figure S1. Hi‐C mapping of eight chromosomes of American lotus. Figure S2. Kimura distance analysis of transposable elements in the American lotus genome. Figure S3. Shared gene families among Arabidopsis thaliana (At), Carica papaya (Cp), Nelumbo lutea (Nl), Nelumbo nucifera (Nn), Oryza sativa (Os), and Vitis vinifera (Vv). Figure S4. Distribution of 67 genes specific to American lotus on eight American lotus chromosomes. Figure S5. Size distribution of structure variants between American and Asian lotus detected using Assemblytics. Figure S6. Size distribution of structure variants between American and Asian lotus detected using assemblytics. Figure S7. Non‐anthocyanin flavonoids and xanthophylls in American and Asian lotus. Figure S8. Phylogenetic tree of MYB genes involved in flavonoid biosynthesis in Amborella trichopoda (Am), Arabidopsis thaliana (At), Nelumbo lutea (Nl), Nelumbo nucifera (Nn), Vitis vinifera (Vv). Figure S9. Thermogenesis in floral receptacles during different stages of flowering. Figure S10. A phylogenetic tree based on the amino acid sequences of UCPs from plants including Arabidopsis thaliana, Mangifera indica, Nelumbo nucifera, Oryza sativa, Phodopus sungorus, Solanum lycopersicum and Zea mays, as well as other animal UCPs from Bos taurus, Canis lupus familiaris, Cyprinus carpio, Danio rerio, Dicrostonyx groenlandicus, Drosophila melanogaster, Homo sapiens, Mus musculus, Mesocricetus auratus, Ochotona dauurica, Oryctolagus cuniculus, Ovis aries, Pongo abelii, Rattus norvegicus, Suncus murinus and Sus scrofa. Figure S11. DEGs in carpels identified by comparing each time point with the previous time point. Figure S12. Geographic distributions and components of lotus accessions. Figure S13. Sequencing depth and heterozygosity of 240 lotus accessions. Figure S14. Venn diagram of SNP number between different lotus groups. Figure S15. Population divergence and nucleotide diversity of different lotus subgroups. Figure S16. Selection in cultivate [file TPJ-110-1498-s001.pdf]

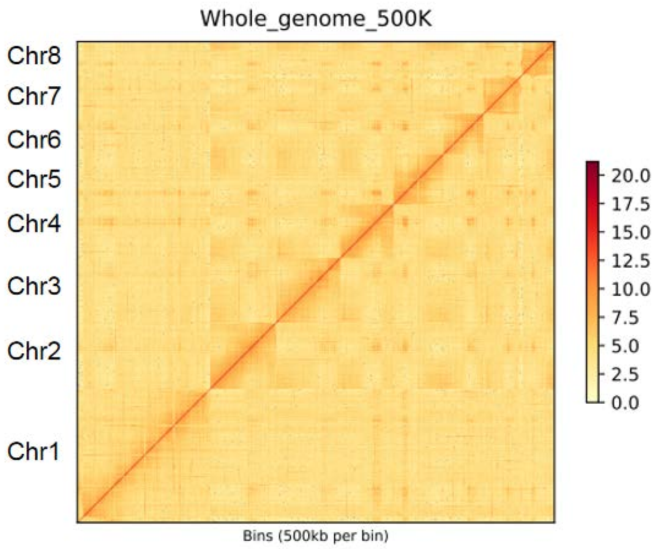


**Figure S1**. **Hi-C mapping of eight chromosomes of American lotus.** Hi-C heat maps are shown at 500-kb resolution.

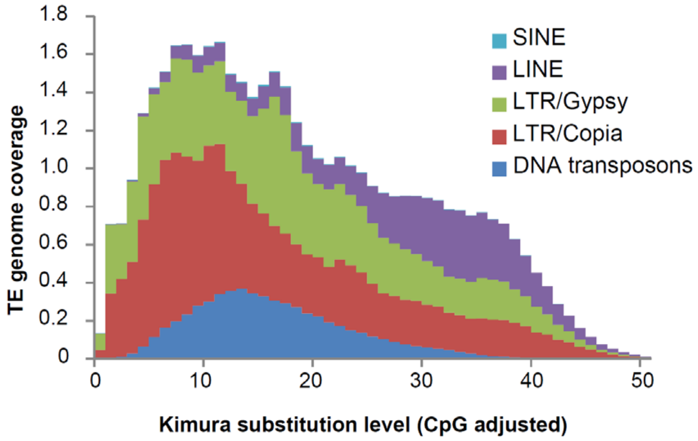


**Figure S2. Kimura distance analysis of transposable elements in the American lotus genome**. The *y*-axis represents genome coverage for each type of TE, including SINE, LINE, *Gypsy* LTR, *Copia* LTR, and DNA transposons.

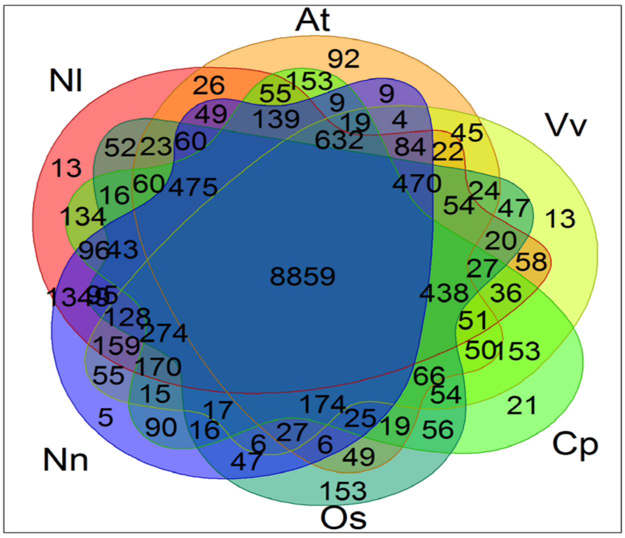


**Figure S3. Shared gene families among *A. thaliana* (At), *O. sativa* (Os), *C. papaya* (Cp), *V. vinifera* (Vv), *N. nucifera* (Nn) and *N. lutea* (Nl).** These six plant species contain 8859 common gene families, and N. lutea has 13 species-specific gene families.

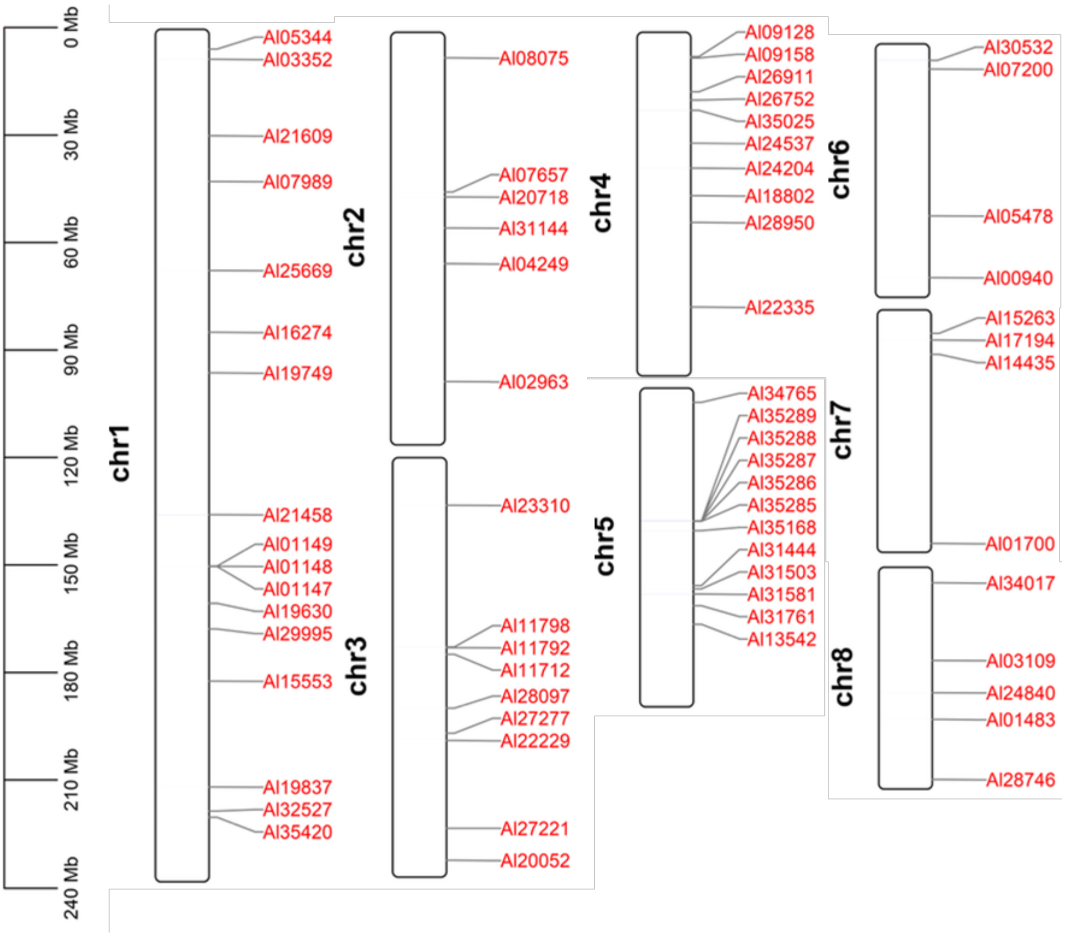


**Figure S4. Distribution of 67 genes specific to American lotus on eight American lotus chromosomes.**

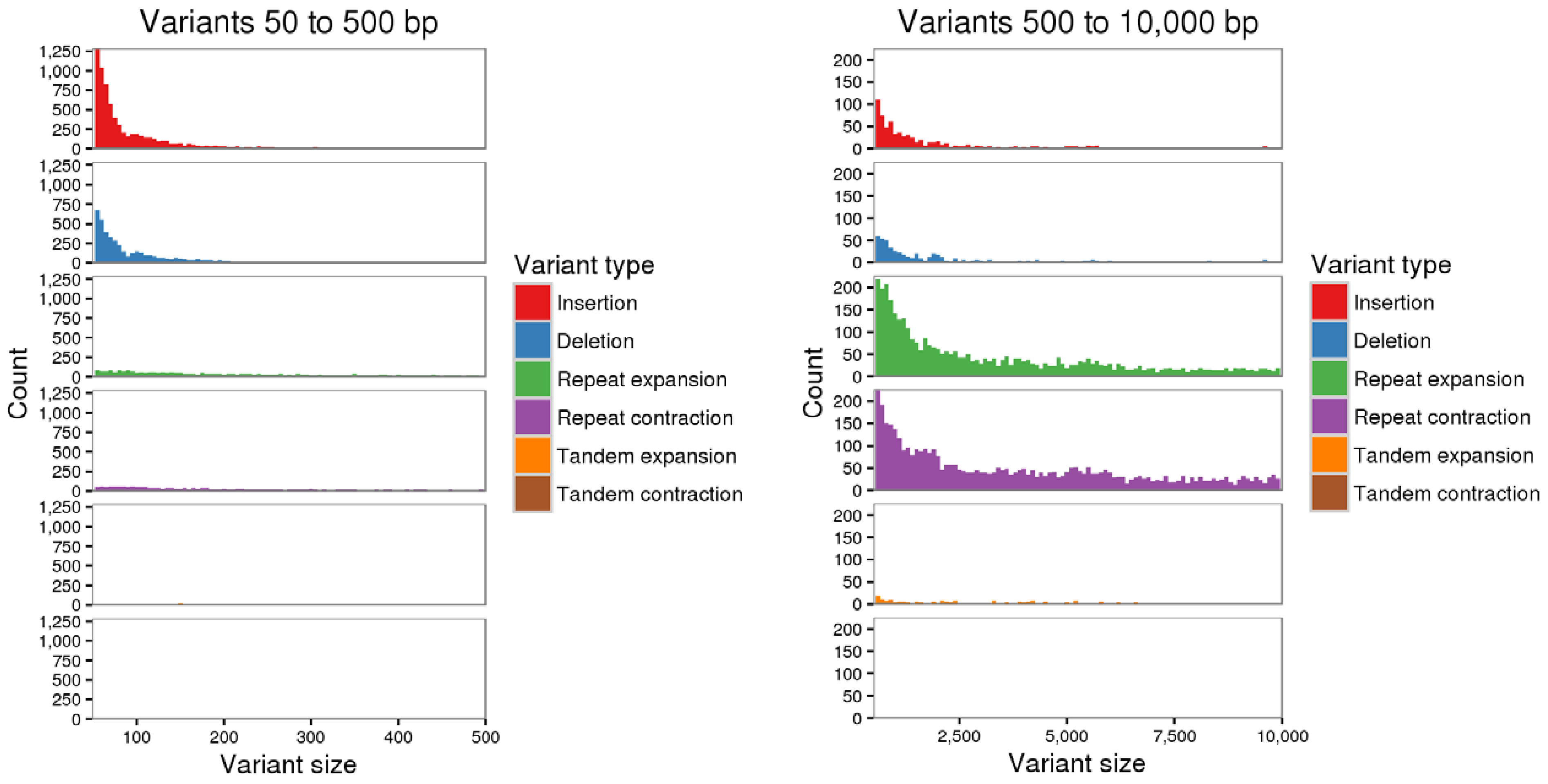


**Figure S5. Size distribution of structure variants between American and Asian lotus detected using Assemblytics.** The left figure displays the distribution of small SVs ranging from 50 to 500 bp. The right figure displays the distribution of large SVs ranging from 500 to 10,000 bp.

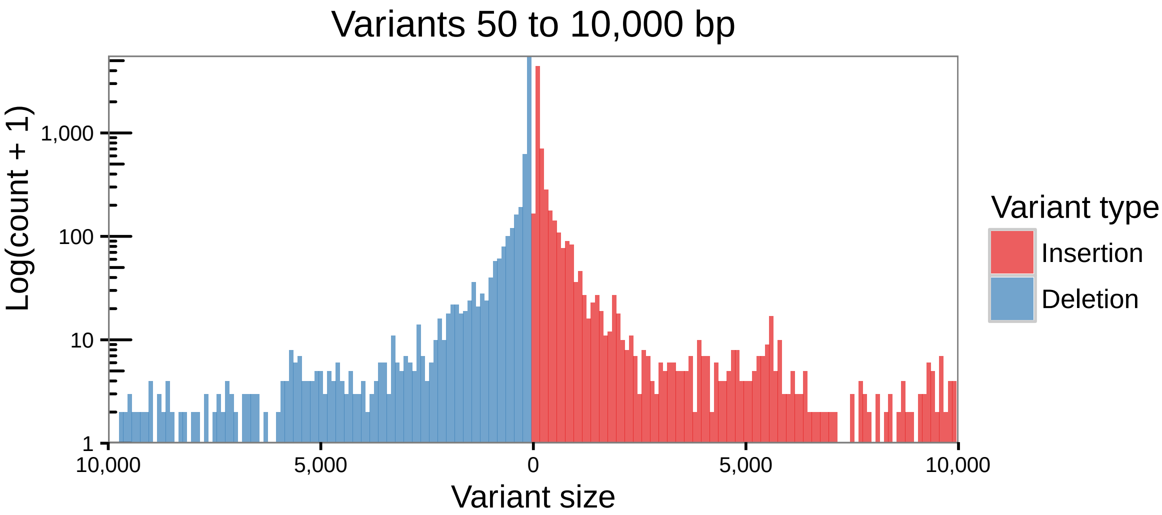


**Figure S6. Size distribution of structure variants between American lotus and Asian lotus detected by Assemblystics.** The genome of Asian lotus was used as reference to detect structure variants.

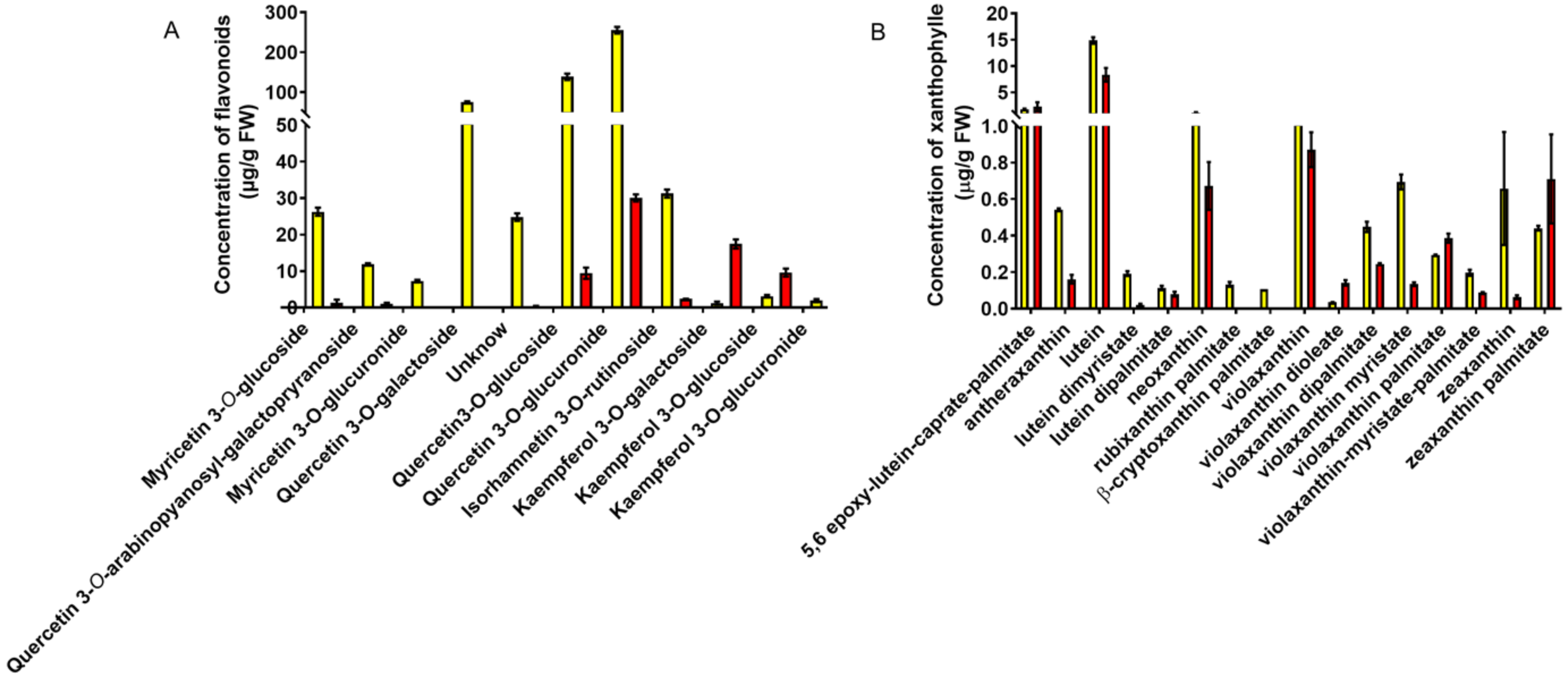


**Figure S7. Non-anthocyanin flavonoids and xanthophylls in American and Asian lotus.** (A) Concentrations of non-anthocyanin flavonoids. (B) Concentrations of xanthophylls. Yellow and red bars represent non-anthocyanin flavonoids and xanthophylls concentrations in American and Asian lotus, respectively.

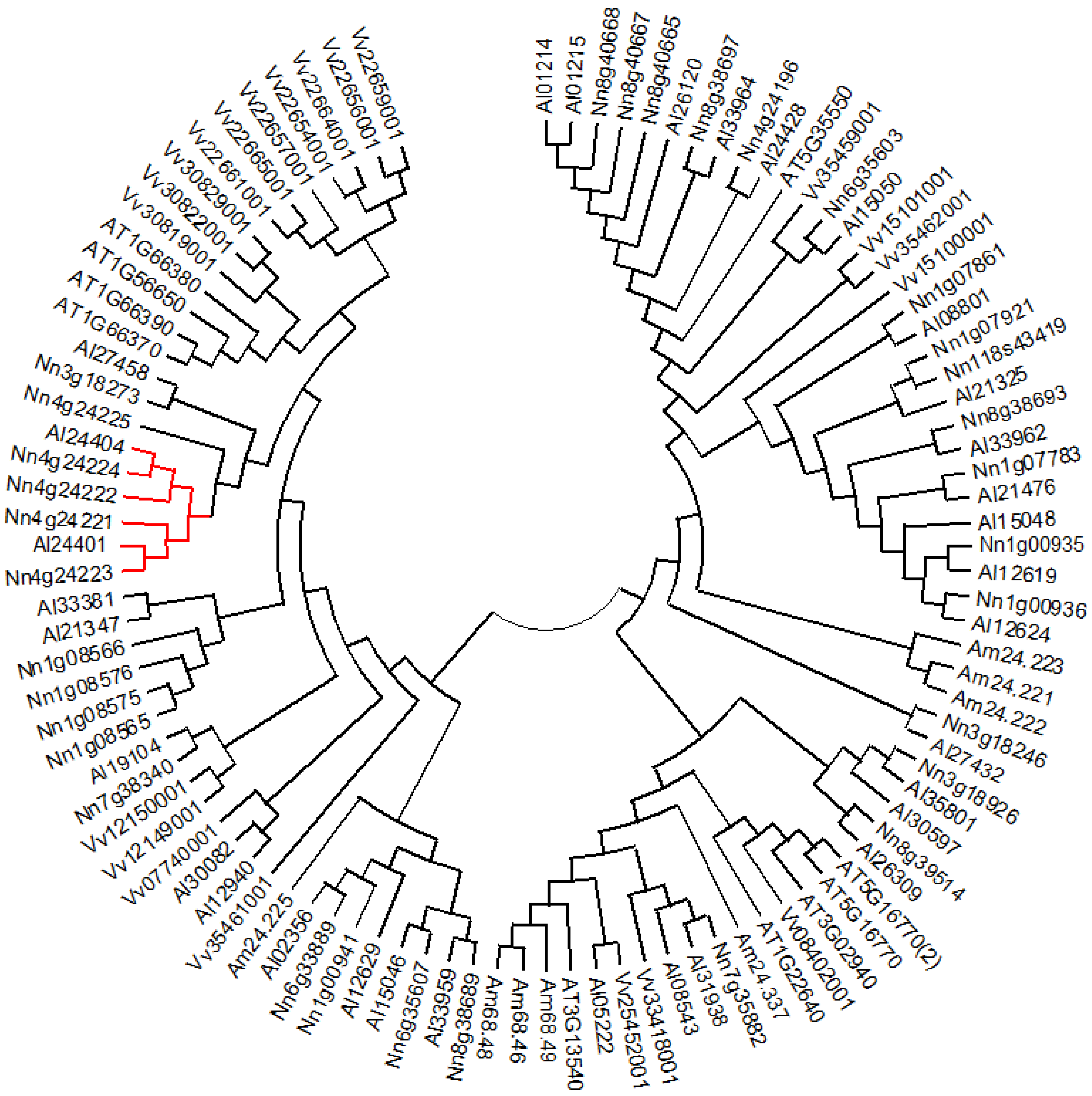


**Figure S8. Phylogenetic tree of *MYB* genes involved in flavonoid biosynthesis in *A. thaliana* (AT), *V. vinifera* (Vv), *A. trichopoda* (Am), *N. nucifera* (Nn), and *N. lutea* (Al).** A cluster of anthocyanin biosynthesis MYB-encoding genes that underwent expansion on chromosome 4 in *N. nucifera* are highlighted in red. The phylogenetic tree was constructed in MEGA X using the maximum likelihood method.

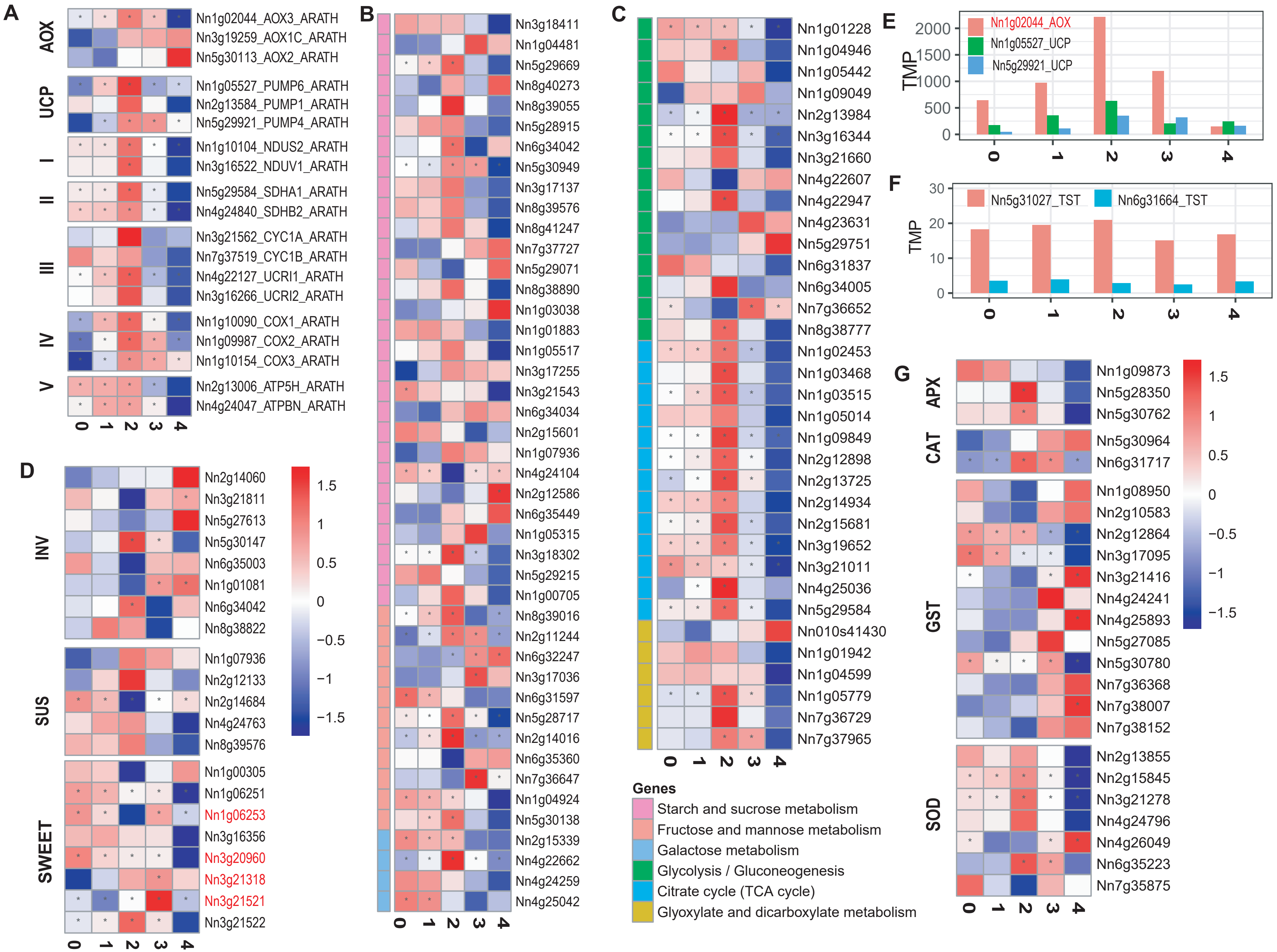


**Figure S9. Thermogenesis in floral receptacles of during different stages of flowering.** Heat map of the transcript abundances of genes with putative functions in (A) cell respiration; (B and C) sugar metabolism, and (D) INVs, SUSs, and SWEETs. INVs, sucrose invertase genes; SUSs, sucrose synthase genes; SWEETs, 'sugars will eventually be exported transporters'. (E) Transcript abundances of AOX and UCPs (only showing the most abundant candidates). (F) TSTs (tonoplastic sugar transporters). (G) Heat map of the transcript abundances of genes encoding antioxidant response related enzymes. Numerals 0-4 indicate stage 0 through stage 4 of flower development with reference to Grant et al., (2008).

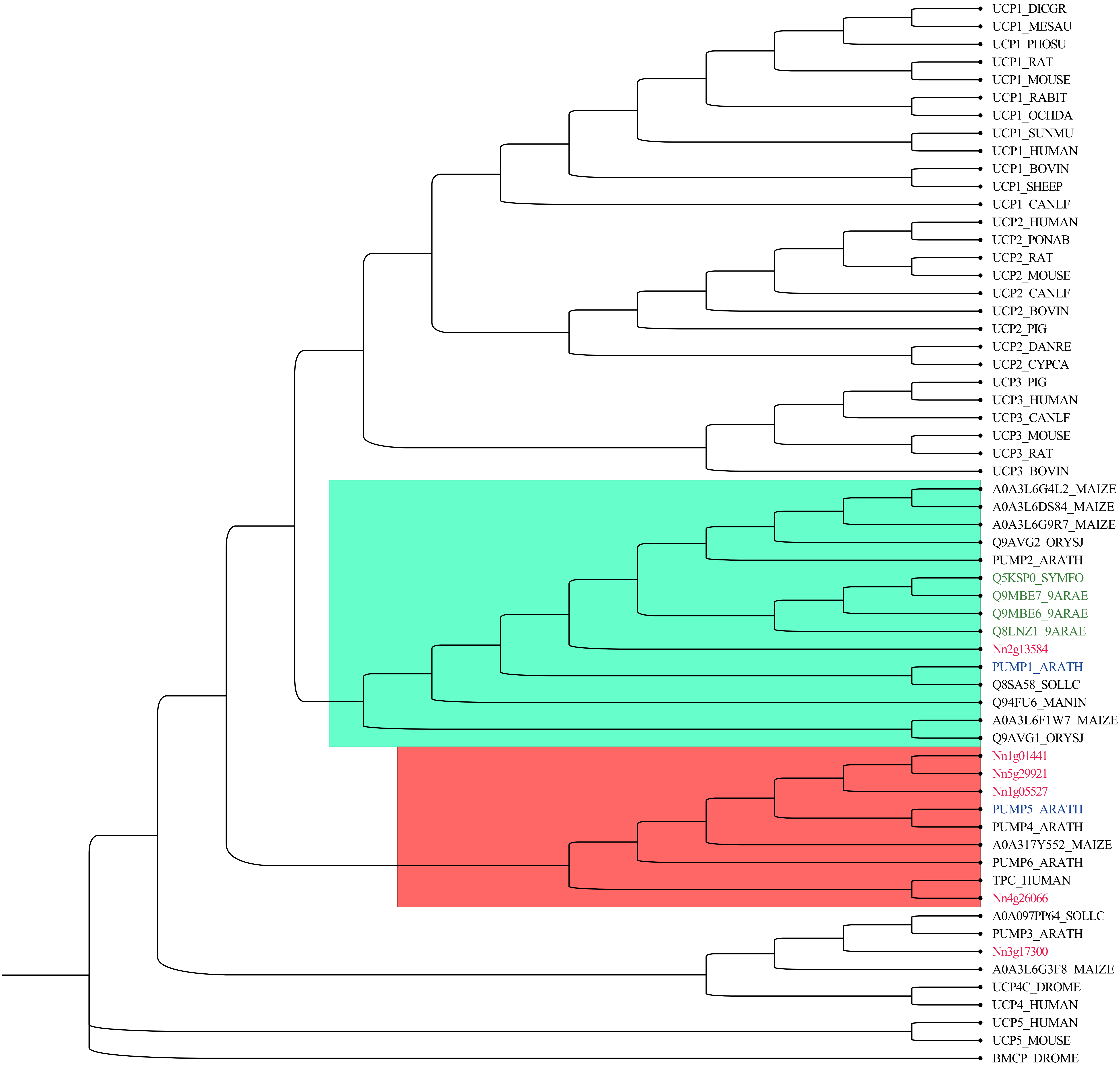


**Figure S10. A phylogenetic tree based on the amino acid sequences of UCPs from plants including *N. nucifera*, *A. thaliana*, *O. sativa*, *Zea mays, Solanum lycopersicum*, *Mangifera indica* and *Phodopus sungorus*, as well as other animal UCPs from *Homo sapiens*, *Drosophila melanogaster*, *Mus musculus*, *Rattus norvegicus*, *Dicrostonyx groenlandicus*, *Suncus murinus*, *Sus scrofa*, *Ovis aries*, *Pongo abelii*, *Danio rerio*, *Cyprinus carpio*, *Mesocricetus auratus*, *Ochotona dauurica*, *Oryctolagus cuniculus*, *Canis lupus familiaris*, and *Bos taurus*.** Red tip labels highlight the six lotus UCPs, blue tip labels highlight AtPUMP1 and AtPUMP5, green tip labels highlight the reported UCPs in thermogenic plants including eastern skunk cabbage *Symplocarpus foetidus*, Asian skunk cabbage *Symplocarpus renifolius*, and the dead horse Arum (*Helicodiceros muscivorus*).

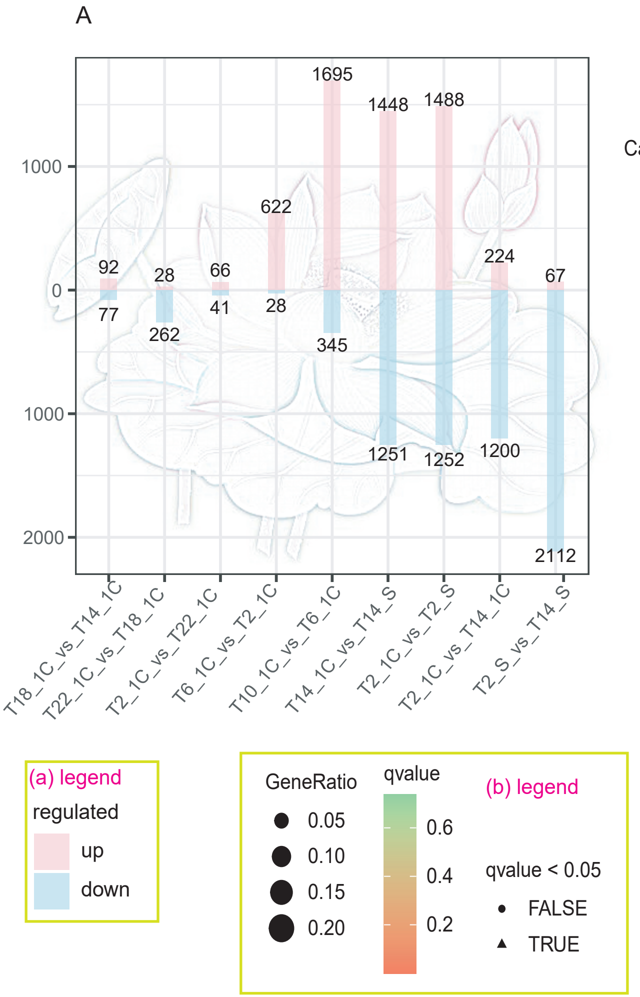


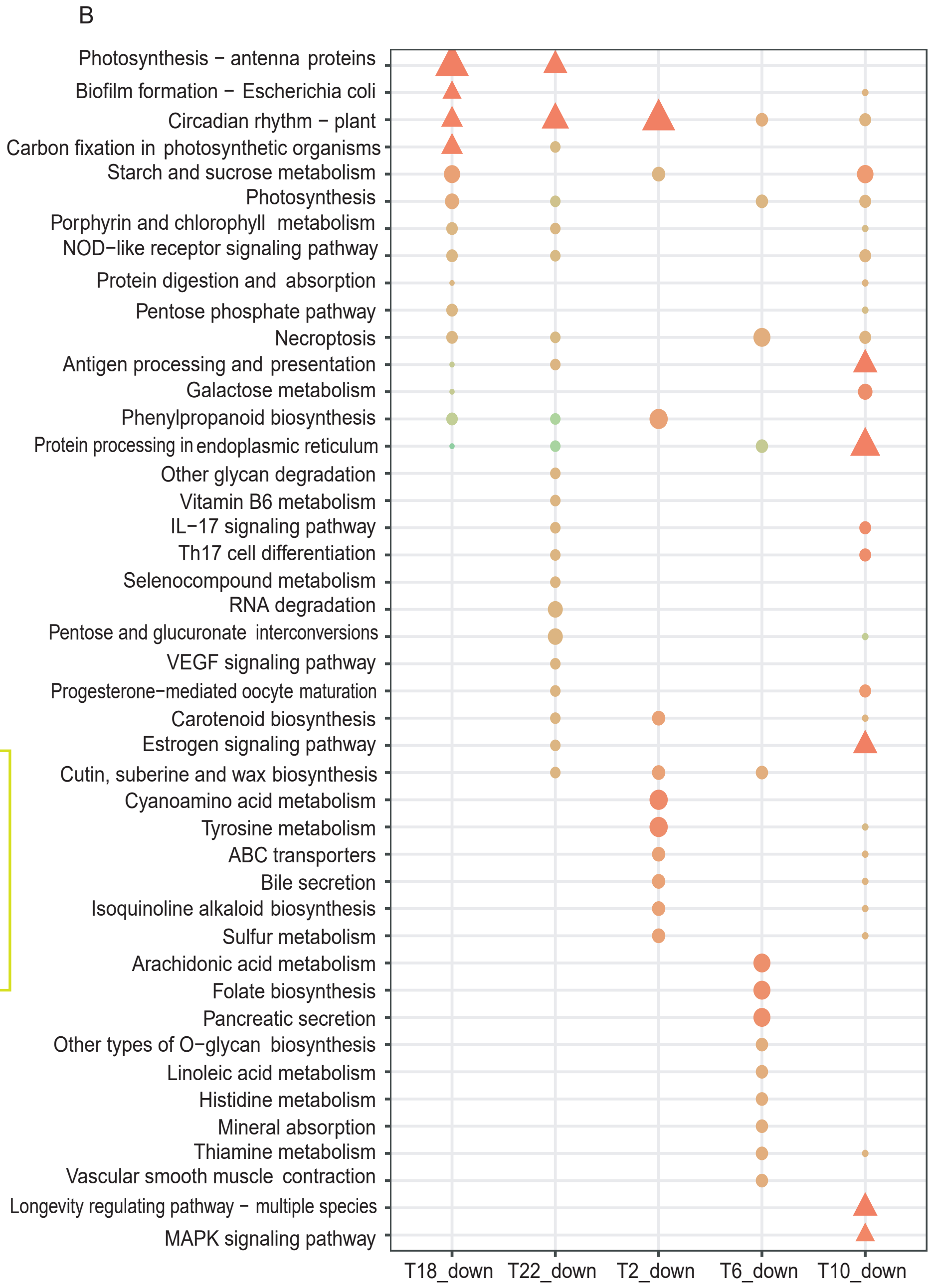


**Figure S11. DEGs in carpels identified by comparing each time point with the previous time point.** (A) Numbers of DEGs in different comparison groups. (B) KEGG enrichment of DEGs with decreased expression compared with the previous time point in carpels.

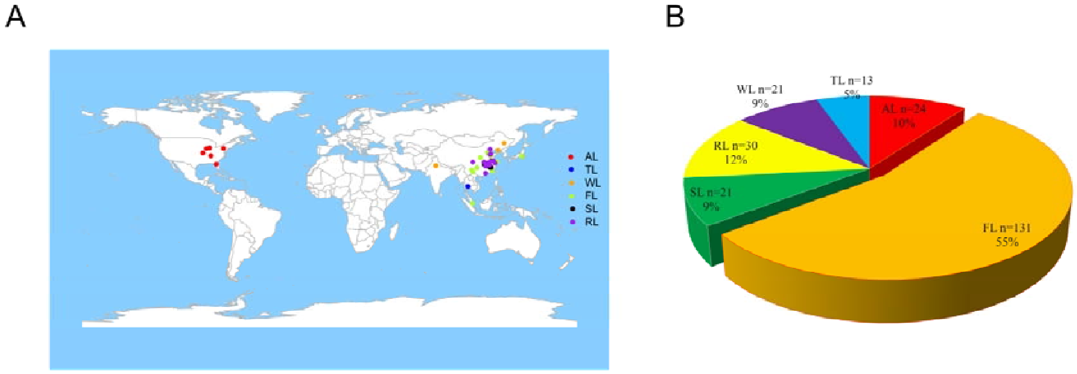


**Figure S12. Geographic distributions (a) and components (b) of lotus accessions.** AL, American lotus accessions; WL, wild Asian lotus accessions; FL, flowering lotus accessions; SL, seed lotus accessions; RL, rhizome lotus accessions; TL, Thailand lotus accessions.

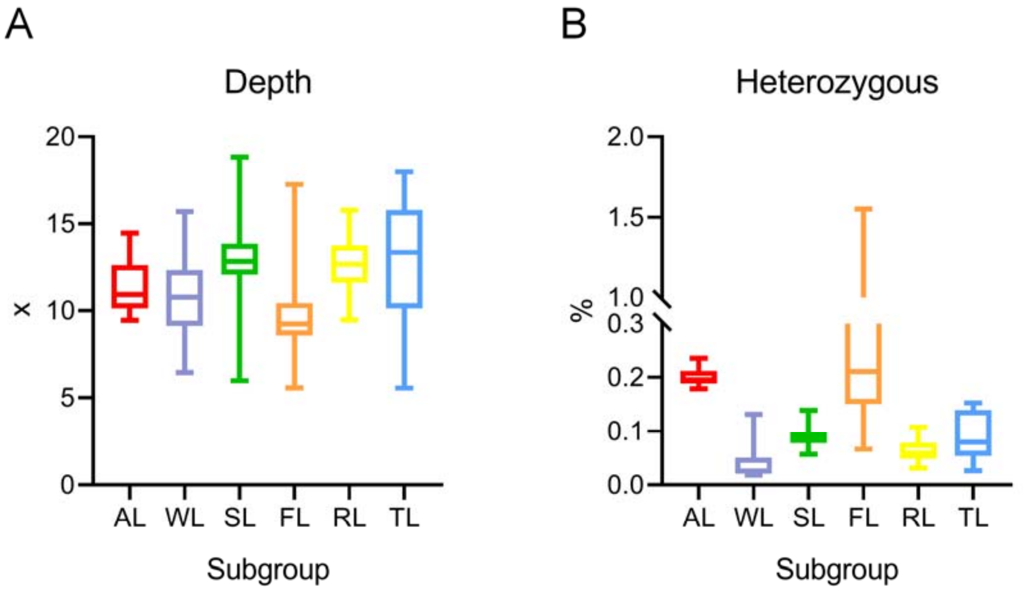


**Figure S13. Sequencing depth (A) and heterozygosity (B) of 240 lotus accessions.**
AL, American lotus accessions; WL, wild Asian lotus accessions; FL, flowering lotus accessions; SL, seed lotus accessions; RL, rhizome lotus accessions; TL, Thailand lotus accessions.

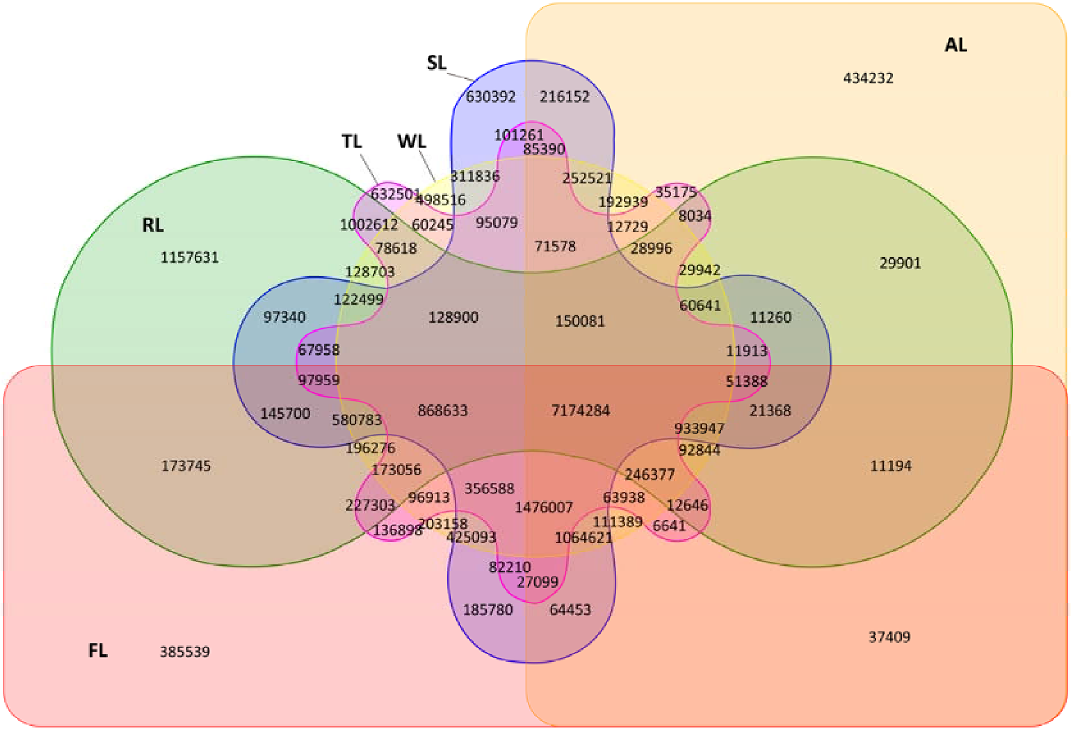


**Figure S14.Venn diagram of SNP number between different lotus groups.** AL, American lotus accessions; WL, wild Asian lotus accessions; FL, flowering lotus accessions; SL, seed lotus accessions; RL, rhizome lotus accessions; TL, Thailand lotus accessions.

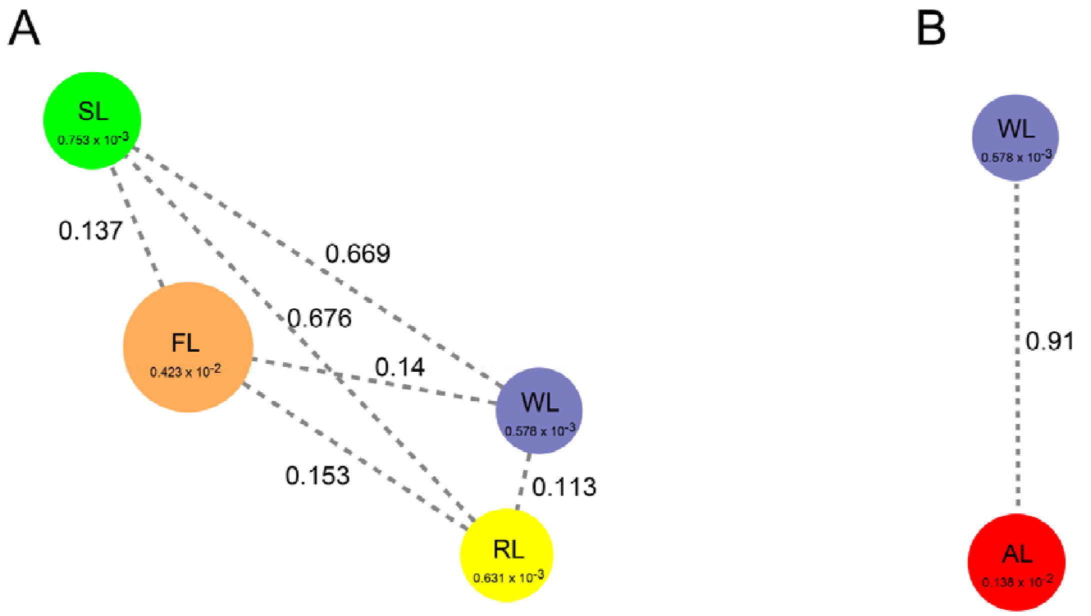


**Figure S15. Population divergence and nucleotide diversity of different lotus subgroups.** (A) Nucleotide diversity (π) and population divergence ($F_{ST}$) across the Asian lotus. The value inside each circle represents the π value of the subgroup, and the value on each line represents the $F_{ST}$ value between subgroups. (B) Population divergence between American lotus (AL) accessions and wild Asian lotus (WL) accessions.

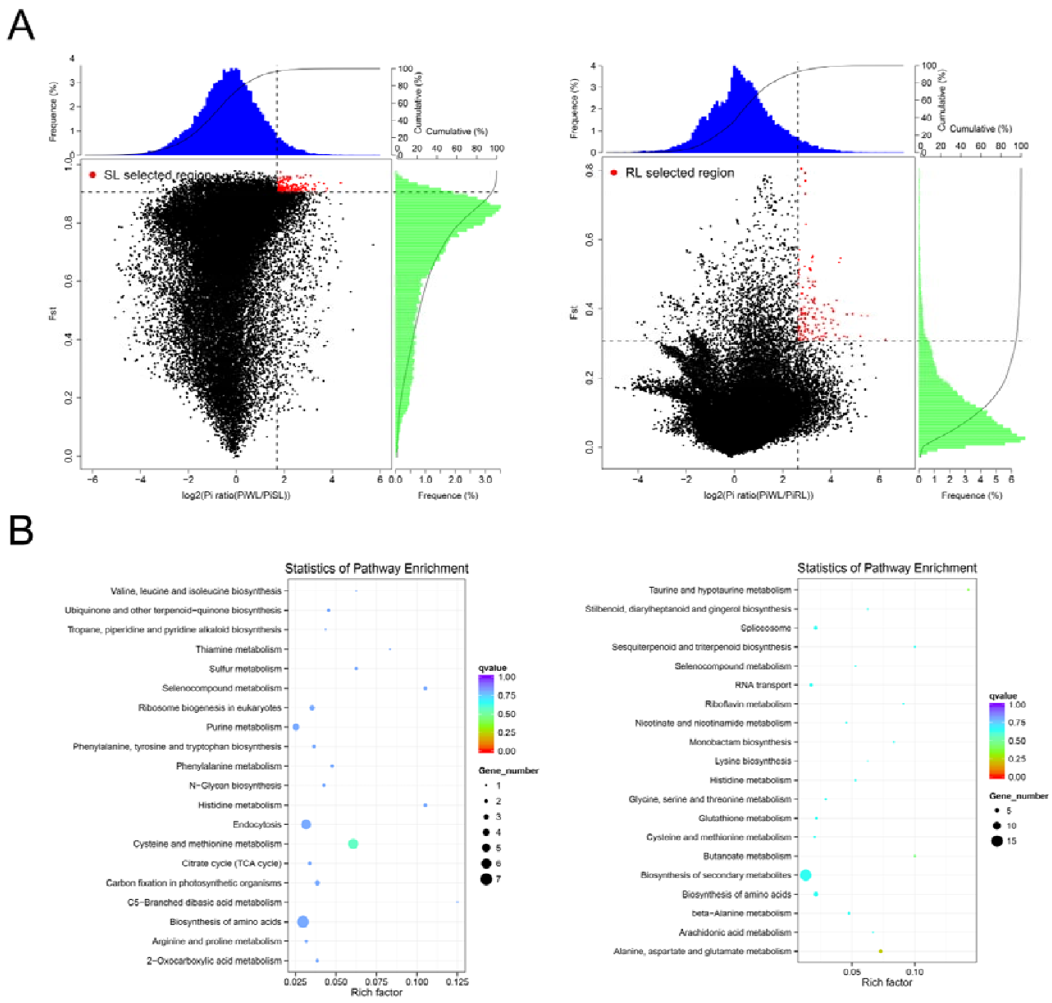


**Figure S16. Selection in cultivated Asian lotus.** (A) Distribution of Pi ratios (Pi π WL/π cultivated lotus) and $F_{ST}$ values. Red dots represent the selective signals in the genomes of SL (seed lotus) (left) and RL (rhizome lotus) (right). (B) KEGG analysis of genes under selection in SL (left) and RL (right), respectively.
