# Supplementary material for: Comparative analyses of American and Asian lotus genomes reveal insights into petal color, carpel thermogenesis and domestication
Source: Plant J. 2022 Apr 19;110(5):1498–515. doi: 10.1111/tpj.15753 (PMC9325450; doi:10.1111/tpj.15753)
Supplement: Supplementary file 2 — Table S1. Sequencing information. Table S2. Statistics of contig‐level genome assembly and annotation. Table S3. Chromosome‐scale genome assembly. Table S4. Assessment of genome assemblies based on assembled RNA‐seq transcripts. Table S5. Annotation of transposable elements (TEs). Table S6. BUSCO completeness analysis of genome assembly and annotation. Table S7. Overall orthogroups in Arabidopsis thaliana, Carica papaya, Oryza sativa, Nelumbo lutea, Nelumbo nucifera, and Vitis vinifera. Table S8. Statistics of orthogroups in Arabidopsis thaliana, Carica papaya, Oryza sativa, Nelumbo lutea, Nelumbo nucifera, and Vitis vinifera. Table S9. List of genes specific to Asian lotus or American lotus. Table S10. List of GO terms enriched in Asian lotus‐specific genes. Table S11. Structural variant statistics. Table S12. Genomic distribution of structural variants. Table S13. List of GO terms enriched in genes affected by structure variants. Table S14. Genes in the anthocyanin biosynthesis pathway of American and Asian lotus. Table S15. Genes in the carotenoid biosynthesis pathway of American and Asian lotus. Table S16. List of flavonoid and carotenoid biosynthesis genes with structural variation between American and Asian lotus. Table S17. Genes used for phylogenetic analysis of flavonoid biosynthesis related to MYB genes. Table S18. Summary of SNPs from all varieties of lotus. Table S19. Statistics of common and unique SNPs across different subgroups. Table S20. Statistics of p i and F ST across different subgroups. Table S21. The heterozygosity ratio in each subgroup. Table S22. Genes under selection associated with domestication in seed and rhizome lotus accessions. Table S23. List of primers used for qRT‐PCR in this study. [file TPJ-110-1498-s002.docx]

**Table S1. Sequencing information**

| **Items** | **Sequencing platform** | |
| --- | --- | --- |
|  | Pacbio RSII | Illumina X10 |
| Total Number of reads | 11,183,985 |  |
| Total Number of sequenced Bases (Gb) | 74.6 |  |
| Mean reads length (bp) | 7,163.56 |  |
| N50 (bp) | 11,136 |  |
| Coverage (X) | ~79 |  |

**Table S2. Statistics of contig-level genome assembly and annotation**

| **Items** | **Contig level assembly** **(NextDenovo)** | **After eliminating redundant sequences(Khaper)** | **Polished genome**  **(NextPolish)** |
| --- | --- | --- | --- |
| Assembly size (bp) | 870,660,468 | 844,248,976 | 843,507,555 |
| No. of contigs | 1,805 | 1,652 | 1,652 |
| Maximum length (bp) | 13,463,642 | 13,463,642 | 13,464,101 |
| N90 (bp) | 180,761 | 192,520 | 192,531 |
| N80 (bp) | 372,444 | 396,437 | 395,522 |
| N70 (bp) | 606,203 | 640,916 | 641,738 |
| N60 (bp) | 887,232 | 924,327 | 923,464 |
| N50 (bp) | 1,317,849 | 1,338,552 | 1,337,422 |
| Average length (bp) | 482,360 | 511,046 | 510,597 |

**Table S3. Chromosome-scale genome assembly**

| **Chromsomes/Items** | **Anchored contigs** | **Length(bp)** |
| --- | --- | --- |
| Chr1 | 690 | 233,304,358 |
| Chr2 | 117 | 115,060,389 |
| Chr3 | 158 | 112,912,317 |
| Chr4 | 95 | 94,198,190 |
| Chr5 | 106 | 87,322,898 |
| Chr6 | 121 | 69,686,065 |
| Chr7 | 191 | 66,479,690 |
| Chr8 | 119 | 60,674,879 |
| Total number of contigs | 1,652 |  |
| Total length of contigs |  | 843,507,555 |
| Total number of anchored contgis | 1,597 |  |
| Total length of chromosome level assembly |  | 838,902,301 |
| Number of unanchored contigs | 55 |  |
| Length of unanchored contigs |  | 4,764,154 |
| Anchor rate (%) | 99 |  |

**Table S4. Assessment of genome assemblies based on assembled RNA-Seq transcripts**

|  | Number | Total Length  (bp) | Accuracy  (%) | Bases covered by assembly(%) | Sequences covered by assembly(%) | With >90% sequence in same chromosome | | With >50% sequence in same chromosome | |
| --- | --- | --- | --- | --- | --- | --- | --- | --- | --- |
|  |  |  |  |  |  | Number | Percent | Number | Percent |
| RNA assembled transcripts | 80876 | 171940161 | 99.99 | 100 | 99.99 | 80866 | 99.99 | 80866 | 99.99 |

**Table S5. Annotation of transposable elements (TEs)**

| **Category** | **Length(Mb)** | **% of genome** |
| --- | --- | --- |
| Total repeat fraction | 680.09 | 81 |
| Class I: Retroelement | 411.64 | 49.03 |
| LTR Retrotransposon | 291.43 | 34.71 |
| Ty1/Copia | 131.52 | 15.66 |
| Ty3/Gypsy | 99.77 | 11.88 |
| Other | 60.14 | 7.16 |
| Non-LTR Retrotransposon | 92.40 | 11.00 |
| LINE | 88.31 | 10.52 |
| SINE | 4.08 | 0.49 |
| Unclassified retroelement | 27.81 | 3.31 |
| Class II: DNA Transposon | 91.46 | 10.89 |
| TIR |  |  |
| CMC | 2.15 | 0.26 |
| hAT | 5.19 | 0.62 |
| Mutator | 1.60 | 0.19 |
| Tc1/Mariner | 0.00 | 0.00 |
| PIF/Harbinger | 0.31 | 0.04 |
| Other | 82.20 | 9.79 |
| Helitron | 0.00 | 0.00 |
| Tandem Repeats | 43.19 | 5.14 |
| Unkown | 12.52 | 1.49 |

**Table S6. BUSCO completeness analysis of genome assembly and annotation**

| **Description** | **Genome** | |  | **Annotation** | |
| --- | --- | --- | --- | --- | --- |
|  | Number | Percentage (%) |  | Number | Percentage (%) |
| Complete BUSCOs (C) | 1,508 | 93.5 |  | 1,464 | 90.7 |
| Single-copy BUSCOs (S) | 1,415 | 87.7 |  | 1,386 | 85.9 |
| Duplicated BUSCOs (D) | 93 | 5.8 |  | 78 | 4.8 |
| Fragmented BUSCOs (F) | 34 | 2.1 |  | 87 | 5.4 |
| Missing BUSCOs (M) | 72 | 4.4 |  | 63 | 3.9 |
| Total BUSCOs | 1,614 | 100.0 |  | 1,614 | 100.0 |

**Table S7. Overall orthogroups in *A. thaliana*, V*. vinifera*, *C. papaya*, *O. sativa,* *N. nucifera*, and *N. lutea***

| **Items** | **Number** |
| --- | --- |
| Number of genes | 199,998 |
| Number of genes in orthogroups | 142,454 |
| Number of unassigned genes | 57,544 |
| Percentage of genes in orthogroups | 71.2 |
| Percentage of unassigned genes | 28.8 |
| Number of orthogroups | 15,685 |
| Number of species-specific orthogroups | 297 |
| Number of genes in species-specific orthogroups | 2,076 |
| Percentage of genes in species-specific orthogroups | 1 |
| Mean orthogroup size | 9.1 |
| Median orthogroup size | 8 |
| G50 (assigned genes) | 10 |
| G50 (all genes) | 8 |
| O50 (assigned genes) | 4,075 |
| O50 (all genes) | 7,267 |
| Number of orthogroups with all species present | 8,859 |
| Number of single-copy orthogroups | 1,176 |

**Table S8. Statistics of orthogroups in in *A. thaliana*, V*. vinifera*, *C. papaya*, *O. sativa,* *N. nucifera*, and *N. lutea***

|  | ***N. lutea*** | ***A. thaliana*** | ***V. vinifera*** | ***C. papaya*** | ***O. sativa*** | ***N. nucifera*** |
| --- | --- | --- | --- | --- | --- | --- |
| Number of genes | 31,382 | 35,386 | 26,346 | 27,775 | 52,424 | 26,685 |
| Number of genes in orthogroups | 26,165 | 27,640 | 19,270 | 19,273 | 27,593 | 22,513 |
| Number of unassigned genes | 5,217 | 7,746 | 7,076 | 8502 | 24,831 | 4,172 |
| Percentage of genes in orthogroups | 83.4 | 78.1 | 73.1 | 69.4 | 52.6 | 84.4 |
| Percentage of unassigned genes | 16.6 | 21.9 | 26.9 | 30.6 | 47.4 | 15.6 |
| Number of orthogroups containing species | 14,167 | 12,267 | 12,250 | 12,441 | 11,879 | 13,605 |
| Percentage of orthogroups containing species | 90.3 | 78.2 | 78.1 | 79.3 | 75.7 | 86.7 |
| Number of species-specific orthogroups | 13 | 92 | 13 | 21 | 153 | 5 |
| Number of genes in species-specific orthogroups | 67 | 525 | 131 | 127 | 1197 | 29 |
| Percentage of genes in species-specific orthogroups | 0.2 | 1.5 | 0.5 | 0.5 | 2.3 | 0.1 |

**Table S9. List of genes specific to Asian lotus or American lotus**

| ***N. nucifera* species-specific gene ID** |
| --- |
| Nn1g00004, Nn1g00081, Nn1g00122, Nn1g00123, Nn1g00240, Nn1g00341, Nn1g00386, Nn1g00393, Nn1g00680, Nn1g00841, Nn1g01057, Nn1g01156, Nn1g01293, Nn1g01431, Nn1g01479, Nn1g01530, Nn1g01655, Nn1g01656, Nn1g01660, Nn1g01664, Nn1g01677, Nn1g01693, Nn1g01695, Nn1g01698, Nn1g01704, Nn1g01707, Nn1g01717, Nn1g01721, Nn1g01725, Nn1g01731, Nn1g01738, Nn1g01739, Nn1g01740, Nn1g01741, Nn1g01747, Nn1g01749, Nn1g01753, Nn1g01761, Nn1g01763, Nn1g01764, Nn1g01766, Nn1g01768, Nn1g01769, Nn1g01773, Nn1g01775, Nn1g01776, Nn1g01781, Nn1g01786, Nn1g01790, Nn1g01796, Nn1g01807, Nn1g01813, Nn1g01821, Nn1g01822, Nn1g01829, Nn1g01833, Nn1g01836, Nn1g01844, Nn1g01846, Nn1g01848, Nn1g01855, Nn1g01859, Nn1g01861, Nn1g01865, Nn1g01867, Nn1g01870, Nn1g01890, Nn1g01907, Nn1g01908, Nn1g01911, Nn1g01914, Nn1g01933, Nn1g01934, Nn1g01937, Nn1g01945, Nn1g01960, Nn1g01963, Nn1g01962, Nn1g01975, Nn1g02078, Nn1g02150, Nn1g02161, Nn1g02395, Nn1g02484, Nn1g02513, Nn1g02524, Nn1g02634, Nn1g02645, Nn1g02777, Nn1g02827, Nn1g02918, Nn1g02971, Nn1g03033, Nn1g03048, Nn1g03068, Nn1g03079, Nn1g03320, Nn1g03328, Nn1g03350, Nn1g03591, Nn1g03801, Nn1g04107, Nn1g04116, Nn1g04117, Nn1g04122, Nn1g04124, Nn1g04157, Nn1g04160, Nn1g04178, Nn1g04344, Nn1g04403, Nn1g04622, Nn1g04632, Nn1g04874, Nn1g05086, Nn1g05295, Nn1g05304, Nn1g05308, Nn1g05325, Nn1g05331, Nn1g05461, Nn1g05787, Nn1g05843, Nn1g06058, Nn1g06059, Nn1g06099, Nn1g06315, Nn1g06319, Nn1g06320, Nn1g06604, Nn1g06948, Nn1g07053, Nn1g07084, Nn1g07160, Nn1g07211, Nn1g07369, Nn1g07448, Nn1g07712, Nn1g07713, Nn1g07718, Nn1g07805, Nn1g07806, Nn1g07925, Nn1g07997, Nn1g08071, Nn1g08162, Nn1g08163, Nn1g08166, Nn1g08248, Nn1g08280, Nn1g08601, Nn1g08770, Nn1g08801, Nn1g08802, Nn1g08804, Nn1g08807, Nn1g08867, Nn1g08873, Nn1g08894, Nn1g08896, Nn1g08956, Nn1g08999, Nn1g09022, Nn1g09447, Nn1g09681, Nn1g09699, Nn1g09762, Nn1g09831, Nn1g09850, Nn2g10518, Nn2g10636, Nn2g10789, Nn2g10790, Nn2g10815, Nn2g10816, Nn2g10834, Nn2g10845, Nn2g10934, Nn2g11034, Nn2g11253, Nn2g11281, Nn2g11349, Nn2g11479, Nn2g11785, Nn2g12146, Nn2g12803, Nn2g12806, Nn2g13501, Nn2g13737, Nn2g13746, Nn2g13794, Nn2g13797, Nn2g13939, Nn2g13944, Nn2g14065, Nn2g14085, Nn2g14271, Nn2g14277, Nn2g14572, Nn2g14577, Nn2g14635, Nn2g14636, Nn2g14689, Nn2g14783, Nn2g14787, Nn2g14803, Nn2g14804, Nn2g14872, Nn2g14873, Nn2g14923, Nn2g14997, Nn2g15017, Nn2g15061, Nn2g15062, Nn2g15103, Nn2g15110, Nn2g15222, Nn2g15247, Nn2g15316, Nn2g15319, Nn2g15378, Nn2g15588, Nn2g15701, Nn2g15771, Nn2g15863, Nn3g16177, Nn3g16249, Nn3g16316, Nn3g16613, Nn3g16637, Nn3g16791, Nn3g16795, Nn3g17184, Nn3g17498, Nn3g17553, Nn3g17915, Nn3g17930, Nn3g18033, Nn3g18110, Nn3g18231, Nn3g18416, Nn3g18656, Nn3g18822, Nn3g18878, Nn3g18906, Nn3g19068, Nn3g19138, Nn3g19153, Nn3g19180, Nn3g19260, Nn3g19331, Nn3g19605, Nn3g19754, Nn3g19883, Nn3g19931, Nn3g19938, Nn3g19949, Nn3g19998, Nn3g20090, Nn3g20163, Nn3g20212, Nn3g20299, Nn3g20300, Nn3g20365, Nn3g20377, Nn3g20378, Nn3g20433, Nn3g20435, Nn3g20660, Nn3g20666, Nn3g20681, Nn3g20788, Nn3g20789, Nn3g20790, Nn3g20795, Nn3g20796, Nn3g20847, Nn3g20855, Nn3g20992, Nn3g21026, Nn3g21071, Nn3g21302, Nn3g21371, Nn3g21382, Nn3g21402, Nn3g21779, Nn3g21875, Nn4g22272, Nn4g22274, Nn4g22522, Nn4g22559, Nn4g22581, Nn4g22681, Nn4g22764, Nn4g22779, Nn4g22859, Nn4g22917, Nn4g23489, Nn4g23490, Nn4g23562, Nn4g23628, Nn4g23840, Nn4g23998, Nn4g24007, Nn4g24063, Nn4g24100, Nn4g24166, Nn4g24293, Nn4g24332, Nn4g24389, Nn4g24431, Nn4g24481, Nn4g24484, Nn4g24587, Nn4g24714, Nn4g24716, Nn4g24735, Nn4g24772, Nn4g24791, Nn4g25064, Nn4g25071, Nn4g25072, Nn4g25120, Nn4g25282, Nn4g25297, Nn4g25347, Nn4g25392, Nn4g25451, Nn4g25523, Nn4g25649, Nn4g25666, Nn4g25674, Nn4g25990, Nn4g26028, Nn4g26313, Nn4g26340, Nn4g26351, Nn4g26505, Nn4g26603, Nn4g26689, Nn5g26720, Nn5g26973, Nn5g27079, Nn5g27138, Nn5g27141, Nn5g27203, Nn5g27378, Nn5g27401, Nn5g27447, Nn5g27516, Nn5g27517, Nn5g27544, Nn5g27663, Nn5g27740, Nn5g27786, Nn5g27787, Nn5g27791, Nn5g27808, Nn5g27946, Nn5g28027, Nn5g28076, Nn5g28089, Nn5g28139, Nn5g28245, Nn5g28298, Nn5g28328, Nn5g28396, Nn5g28461, Nn5g28618, Nn5g28661, Nn5g28662, Nn5g28663, Nn5g28690, Nn5g28765, Nn5g29008, Nn5g29075, Nn5g29230, Nn5g30068, Nn5g30146, Nn5g30406, Nn5g30570, Nn5g30578, Nn5g30720, Nn5g30806, Nn5g30947, Nn5g30954, Nn5g31104, Nn5g31131, Nn5g31132, Nn6g31591, Nn6g31592, Nn6g31613, Nn6g31706, Nn6g31891, Nn6g31892, Nn6g31948, Nn6g31953, Nn6g31974, Nn6g31976, Nn6g32029, Nn6g32105, Nn6g32125, Nn6g32209, Nn6g32280, Nn6g32341, Nn6g32348, Nn6g32398, Nn6g32405, Nn6g32406, Nn6g32408, Nn6g32409, Nn6g32463, Nn6g32828, Nn6g33267, Nn6g33448, Nn6g33583, Nn6g33947, Nn6g33999, Nn6g34332, Nn6g34540, Nn6g34543, Nn6g34545, Nn6g34546, Nn6g34884, Nn6g35059, Nn6g35191, Nn6g35268, Nn6g35288, Nn6g35376, Nn6g35420, Nn6g35613, Nn7g35794, Nn7g35938, Nn7g36036, Nn7g36213, Nn7g36219, Nn7g36422, Nn7g36455, Nn7g36551, Nn7g36626, Nn7g36968, Nn7g37018, Nn7g37161, Nn7g37315, Nn7g37316, Nn7g37408, Nn7g37409, Nn7g37496, Nn7g37518, Nn7g37546, Nn7g37548, Nn7g37549, Nn7g37629, Nn7g37656, Nn7g37704, Nn7g37763, Nn7g37958, Nn7g37968, Nn7g38132, Nn7g38186, Nn7g38252, Nn7g38257, Nn7g38258, Nn7g38273, Nn8g38871, Nn8g38982, Nn8g38984, Nn8g39377, Nn8g39424, Nn8g39615, Nn8g39660, Nn8g39661, Nn8g39778, Nn8g39939, Nn8g39985, Nn8g40061, Nn8g40133, Nn8g40366, Nn8g40473, Nn8g40515, Nn8g40540, Nn8g40546, Nn8g40572, Nn8g40580, Nn8g40611, Nn8g40616, Nn8g40695, Nn8g40730, Nn8g40957, Nn8g40964, Nn8g40978, Nn8g40979, Nn8g40991, Nn003s41373, Nn003s41375, Nn006s41417, Nn011s41454, Nn050s41919, Nn109s43307, Nn127s43702, Nn210s45183 |
| ***N. lutea* species-specific gene ID** |
| Al03337, Al05870, Al06222, Al06223, Al06266, Al06276, Al08272, Al09876, Al10289, Al10575, Al11911, Al14353, Al15319, Al15960, Al16322, Al16655, Al17337, Al19098, Al20649, Al20650, Al20651, Al22370, Al24475, Al25226, Al25227, Al25356, Al25763, Al25990, Al26491, Al26641, Al27129, Al27958, Al28917, Al29262, Al29585, Al30186, Al30998, Al30999, Al32526, Al33866, Al34380, Al34957, Al36198 |

**Table S10. List of GO terms enriched in Asian lotus-specific genes**

| **ID** | **Description** | **GeneRatio** | **BgRatio** | **pvalue** | **p.adjust** | **qvalue** |
| --- | --- | --- | --- | --- | --- | --- |
| GO:0000786 | Cellular Component: nucleosome | 7/79 | 62/15275 | 2.95E-08 | 2.354E-06 | 2.065E-06 |
| GO:0006334 | Biological Process: nucleosome assembly | 7/79 | 65/15275 | 4.13E-08 | 2.354E-06 | 2.065E-06 |
| GO:0003824 | Molecular Function: catalytic activity | 7/79 | 259/15275 | 0.0003808 | 0.0144719 | 0.0126947 |
| GO:0008483 | Molecular Function: transaminase activity | 2/79 | 13/15275 | 1.99E-03 | 0.0526325 | 0.0461689 |
| GO:0015079 | Molecular Function: potassium ion transmembrane transporter activity | 2/79 | 14/15275 | 0.0023084 | 0.0526325 | 0.0461689 |
| GO:0047213 | Molecular Function: anthocyanidin 3-O-glucosyltransferase activity | 2/79 | 17/15275 | 0.0034155 | 0.0648942 | 0.0569247 |
| GO:0016740 | Molecular Function: transferase activity | 5/79 | 210/15275 | 4.63E-03 | 0.0673165 | 0.0590496 |
| GO:0010033 | Biological Process: response to organic substance | 2/79 | 20/15275 | 0.004724 | 0.0673165 | 0.0590496 |
| GO:0031347 | Biological Process: regulation of defense response | 2/79 | 22/15275 | 0.0057051 | 0.0722642 | 0.0633897 |
| GO:0006813 | Biological Process: potassium ion transport | 2/79 | 24/15275 | 0.0067711 | 0.0760205 | 0.0666847 |
| GO:0044723 | Biological Process: single-organism carbohydrate metabolic process | 2/79 | 25/15275 | 0.0073353 | 0.0760205 | 0.0666847 |
| GO:0051707 | Biological Process: response to other organism | 2/79 | 28/15275 | 0.0091505 | 0.0820963 | 0.0720143 |
| GO:0045087 | Biological Process: innate immune response | 2/79 | 29/15275 | 0.0097956 | 0.0820963 | 0.0720143 |
| GO:0004674 | Molecular Function: protein serine/threonine kinase activity | 7/79 | 464/15275 | 0.010082 | 0.0820963 | 0.0720143 |
| GO:0016301 | Molecular Function: kinase activity | 4/79 | 197/15275 | 0.019033 | 0.1446505 | 0.1268864 |
| GO:1901700 | Biological Process: response to oxygen-containing compound | 2/79 | 43/15275 | 0.020796 | 0.1457315 | 0.1278346 |
| GO:0016023 | Cellular Component: cytoplasmic membrane-bounded vesicle | 3/79 | 115/15275 | 0.0217319 | 0.1457315 | 0.1278346 |
| GO:0009826 | Biological Process: unidimensional cell growth | 2/79 | 51/15275 | 0.0285959 | 0.1811077 | 0.1588664 |

**Table S11. Structural variant statistics.** Whole genomes of Asian lotus or American lotus were aligned using MUMumer 4.0, and then structural variants were retrieved using online analytics tool Assemblytics.

| **Size of SVs** | **Insertion** | | **Deletion** | | **Tandem_expansion** | | **Tandem_contraction** | | **Repeat_expansion** | | **Repeat_contraction** | |
| --- | --- | --- | --- | --- | --- | --- | --- | --- | --- | --- | --- | --- |
|  | **Count** | **Total bp** | **Count** | **Total bp** | **Count** | **Total bp** | **Count** | **Total bp** | **Count** | **Total bp** | **Count** | **Total bp** |
| 50-500 bp: | 8,301 | 831,631 | 5,105 | 539,211 | 407 | 86,276 | 37 | 8,010 | 2,955 | 629,883 | 2,338 | 496,267 |
| 500-10,000 bp: | 859 | 1,695,202 | 627 | 1,477,131 | 304 | 1,230,339 | 19 | 89,959 | 4,009 | 12,619,128 | 4,572 | 16,754,346 |
| Total: | 9,160 | 2,526,833 | 5,732 | 2,016,342 | 711 | 1,316,615 | 56 | 97,969 | 6,964 | 13,249,011 | 6,910 | 17,250,613 |

Total number of structural variants: 29,533

Total bases affected by structural variants: 36.46 Mbp

| **Table S12. Genomic distribution of structural variants** | | |
| --- | --- | --- |
| **%** | **Number** | **Region** |
| 3.59597738 | 1062 | downstream |
| 4.26641384 | 1260 | exonic |
| 62.4623303 | 18447 | intergenic |
| 23.0318627 | 6802 | intronic |
| 0.04401856 | 13 | splicing |
| 3.81268412 | 1126 | upstream |
| 0.23702299 | 70 | upstream;downstream |
| 1.45599837 | 430 | UTR3 |
| 1.05305929 | 311 | UTR5 |
| 0.04063251 | 12 | UTR5;UTR3 |
| 100 | 29533 |  |

**Table S13. List of GO terms enriched in genes affected by structure variants**

| **GO term** | **Description** | **GeneRatio** | **BgRatio** | **pvalue** | **p.adjust** | **qvalue** |
| --- | --- | --- | --- | --- | --- | --- |
| GO:0007346 | Biological Process: regulation of mitotic cell cycle | 11/3433 | 15/15275 | 3.98E-05 | 0.0227658 | 0.0216621 |
| GO:0009560 | Biological Process: embryo sac egg cell differentiation | 22/3433 | 44/15275 | 5.66E-05 | 0.0227658 | 0.0216621 |
| GO:0006396 | Biological Process: RNA processing | 30/3433 | 70/15275 | 0.0001125 | 0.0227658 | 0.0216621 |
| GO:0004222 | Molecular Function: metalloendopeptidase activity | 19/3433 | 37/15275 | 1.14E-04 | 0.0227658 | 0.0216621 |
| GO:0009793 | Biological Process: embryo development ending in seed dormancy | 63/3433 | 182/15275 | 0.0001151 | 0.0227658 | 0.0216621 |
| GO:0008565 | Molecular Function: protein transporter activity | 19/3433 | 39/15275 | 0.0002778 | 0.0409535 | 0.038968 |
| GO:0009909 | Biological Process: regulation of flower development | 39/3433 | 103/15275 | 2.90E-04 | 0.0409535 | 0.038968 |

**Table S14. Genes in the anthocyanin biosynthesis pathway between of American and Asian lotus.** Reads counts represent the expression of those genes in the two species identified from Iso-Seq libraries prepared from mixed tissues of each species.

| **Gene** | ***N. lutea*** | **Reads count** | ***N. nucifera*** | **Reads count** |
| --- | --- | --- | --- | --- |
| CHS | Al11427 | 0 | Nn3g19912 | 0 |
|  | Al11422 | 76 | Nn3g19909 | 0 |
|  | Al11425 | 0 |  |  |
|  | Al11419 | 0 |  |  |
| CHI | Al17932 | 0 | Nn2g10310 | 23 |
|  | Al20681 | 0 | Nn2g10542 | 1 |
|  | Al22104 | 10 | Nn2g12834 | 24 |
|  | Al25455 | 0 | Nn5g26853 | 9 |
|  | Al34791 | 14 |  |  |
| F3H | Al15781 | 21 | Nn5g28145 | 61 |
| F3'H | Al22974 | 0 | Nn3g17431 | 30 |
|  | Al26732 | 8 | Nn4g23411 | 17 |
| F3'5'H | Al08550 | 9 | Nn2g15849 | 0 |
|  | Al08551 | 1 | Nn3g16486 | 2 |
|  | Al15523 | 0 | Nn7g36756 | 32 |
|  | Al16160 | 0 |  |  |
|  | Al18529 | 0 |  |  |
| DFR | Al10349 | 0 | Nn5g28053 | 0 |
|  | Al10350 | 0 | Nn5g28056 | 0 |
|  | Al10351 | 0 | Nn5g28645 | 1 |
|  | Al10352 | 0 |  |  |
|  | Al12377 | 0 |  |  |
|  | Al31427 | 0 |  |  |
| ANS | Al01937 | 0 | Nn1g01275 | 118 |
|  | Al25964 | 0 | Nn6g34327 | 0 |
|  | Al31279 | 0 |  |  |
|  | Al31280 | 0 |  |  |
| ANGT | Al01581 | 0 | Nn7g36407 | 0 |
|  | Al05142 | 0 | Nn7g36408 | 0 |
|  | Al16055 | 0 | Nn7g36414 | 0 |
|  | Al16056 | 0 | Nn7g36415 | 0 |
|  | Al16058 | 0 | Nn7g36416 | 0 |
|  | Al33517 | 6 | Nn7g36417 | 0 |
|  |  |  | Nn8g40170 | 7 |
|  |  |  | Nn8g40512 | 0 |
|  |  |  | Nn8g40696 | 3 |

**Table S15. Genes in the carotenoid biosynthesis pathway of American and Asian lotus.** Reads counts represent the expression of those genes in the two species identified from Iso-Seq libraries prepared from mixed tissues of each species.

| **Gene** | ***N. lutea*** | **Reads count** | ***N. nucifera*** | **Reads count** |
| --- | --- | --- | --- | --- |
| PSY | Al23953 | 11 | Nn4g24773 | 20 |
|  | Al23903 | 2 | Nn7g38019 | 2 |
|  | Al17470 | 41 | Nn6g32217 | 45 |
|  |  |  | Nn4g25799 | 7 |
| PDS | Al14847 | 1 | Nn6g31492 | 3 |
|  | Al29524 | 1 |  |  |
| Z-ISO | Al35558 | 0 | Nn1g09788 | 2 |
| ZDS | Al26922 | 16 | Nn1g02298 | 16 |
|  | Al26924 | 9 |  |  |
| CISO | Al22532 | 4 | Nn2g14414 | 6 |
|  | Al29744 | 0 | Nn5g27484 | 7 |
| βLCY | Al06813 | 0 | Nn4g26462 | 3 |
|  |  |  | Nn7g36588 | 6 |
| δLCY | Al06979 | 0 |  |  |
|  | Al34049 | 2 | Nn8g38788 | 3 |
| CYP97A3 | Al11452 | 3 | Nn1g03127 | 6 |
|  | Al19361 | 3 | Nn3g19881 | 2 |
| εCH | Al09701 | 9 | Nn2g13887 | 8 |
|  |  |  | Nn6g35182 | 8 |
| βCH | Al02218 | 19 | Nn1g01633 | 11 |
| VDE1 | Al20911 | 2 | Nn1g07946 | 3 |
| ZEP | Al17112 | 12 | Nn5g30293 | 17 |
|  | Al17520 | 2 |  |  |
| CCD |  |  | Nn2g15315 | 13 |
|  | Al21078 | 0 | Nn1g09375 | 1 |
|  | Al12617 | 0 | Nn1g00934 | 1 |
|  | Al34331 | 0 | Nn8g39130 | 0 |
| NSY | Al22594 | 2 | Nn3g17869 | 4 |
|  | Al24700 | 7 | Nn4g23871 | 3 |
|  | Al34089 | 0 |  |  |
| NCED3 | Al06312 | 1 | Nn1g09378 | 3 |
|  | Al07041 | 0 | Nn2g15931 | 3 |
|  | Al15183 | 3 | Nn3g20474 | 6 |
|  | Al21081 | 0 |  |  |

**Table S16. List of flavonoids and carotenoids biosynthesis genes with structural variants between American and Asian lotus**

| **Biosynthesis pathway** | **Gene** | **Gene ID** | **SV location** |
| --- | --- | --- | --- |
| Carotenoids | ZDS | Nn1g02298 | intronic |
|  | AAO | Nn2g14471 | intronic |
|  | CCD | Nn2g15315 | intronic |
|  | CYP97A3 | Nn3g19881 | intronic |
|  | NSY | Nn4g23871 | intronic |
|  | PSY | Nn4g25799 | downstream |
|  | PDS | Nn6g31492 | intronic |
|  | PSY | Nn7g38019 | exonic |
|  | δLCY | Nn8g38788 | intronic |
| Flavonoids / Anthocyanins | F3'H | Nn3g17431 | exonic |
|  | DFR | Nn5g28056 | intronic |
|  | DFR | Nn5g28056 | upstream |
|  | ANGT | Nn7g36416 | exonic |
|  | ANGT | Nn7g38337 | intronic |

**Table S17. Genes used for phylogenetic analysis of flavonoid biosynthesis related to *MYB* genes**

| **Gene** | **Gene ID** | **Species** |
| --- | --- | --- |
| AT1G22640 | AT1G22640 | *A. thaliana* |
| AT1G56650 | AT1G56650 | *A. thaliana* |
| AT1G66370 | AT1G66370 | *A. thaliana* |
| AT1G66380 | AT1G66380 | *A. thaliana* |
| AT1G66390 | AT1G66390 | *A. thaliana* |
| AT3G02940 | AT3G02940 | *A. thaliana* |
| AT3G13540 | AT3G13540 | *A. thaliana* |
| AT5G16770 | AT5G16770 | *A. thaliana* |
| AT5G16770 | AT5G16770 | *A. thaliana* |
| AT5G35550 | AT5G35550 | *A. thaliana* |
| Am24.221 | evm_27.model.AmTr_v1.0_scaffold00024.221 | *A. trichopoda* |
| Am24.222 | evm_27.model.AmTr_v1.0_scaffold00024.222 | *A. trichopoda* |
| Am24.223 | evm_27.model.AmTr_v1.0_scaffold00024.223 | *A. trichopoda* |
| Am24.225 | evm_27.model.AmTr_v1.0_scaffold00024.225 | *A. trichopoda* |
| Am24.337 | evm_27.model.AmTr_v1.0_scaffold00024.337 | *A. trichopoda* |
| Am68.46 | evm_27.model.AmTr_v1.0_scaffold00068.46 | *A. trichopoda* |
| Am68.48 | evm_27.model.AmTr_v1.0_scaffold00068.48 | *A. trichopoda* |
| Am68.49 | evm_27.model.AmTr_v1.0_scaffold00068.49 | *A. trichopoda* |
| Vv07740001 | GSVIVT01007740001 | *V. vinifera* |
| Vv08402001 | GSVIVT01008402001 | *V. vinifera* |
| Vv12149001 | GSVIVT01012149001 | *V. vinifera* |
| Vv12150001 | GSVIVT01012150001 | *V. vinifera* |
| Vv15100001 | GSVIVT01015100001 | *V. vinifera* |
| Vv15101001 | GSVIVT01015101001 | *V. vinifera* |
| Vv22654001 | GSVIVT01022654001 | *V. vinifera* |
| Vv22656001 | GSVIVT01022656001 | *V. vinifera* |
| Vv22657001 | GSVIVT01022657001 | *V. vinifera* |
| Vv22659001 | GSVIVT01022659001 | *V. vinifera* |
| Vv22661001 | GSVIVT01022661001 | *V. vinifera* |
| Vv22664001 | GSVIVT01022664001 | *V. vinifera* |
| Vv22665001 | GSVIVT01022665001 | *V. vinifera* |
| Vv25452001 | GSVIVT01025452001 | *V. vinifera* |
| Vv30819001 | GSVIVT01030819001 | *V. vinifera* |
| Vv30822001 | GSVIVT01030822001 | *V. vinifera* |
| Vv30829001 | GSVIVT01030829001 | *V. vinifera* |
| Vv33418001 | GSVIVT01033418001 | *V. vinifera* |
| Vv35459001 | GSVIVT01035459001 | *V. vinifera* |
| Vv35461001 | GSVIVT01035461001 | *V. vinifera* |
| Vv35462001 | GSVIVT01035462001 | *V. vinifera* |
| Nn7g35882 | Nn7g35882 | *N. nucifera* |
| Nn7g38340 | Nn7g38340 | *N. nucifera* |
| Nn8g38689 | Nn8g38689 | *N. nucifera* |
| Nn8g38693 | Nn8g38693 | *N. nucifera* |
| Nn8g38697 | Nn8g38697 | *N. nucifera* |
| Nn8g39514 | Nn8g39514 | *N. nucifera* |
| Nn8g40665 | Nn8g40665 | *N. nucifera* |
| Nn8g40667 | Nn8g40667 | *N. nucifera* |
| Nn8g40668 | Nn8g40668 | *N. nucifera* |
| Nn3g18246 | Nn3g18246 | *N. nucifera* |
| Nn3g18273 | Nn3g18273 | *N. nucifera* |
| Nn3g18926 | Nn3g18926 | *N. nucifera* |
| Nn4g24196 | Nn4g24196 | *N. nucifera* |
| Nn4g24221 | Nn4g24221 | *N. nucifera* |
| Nn4g24222 | Nn4g24222 | *N. nucifera* |
| Nn4g24223 | Nn4g24223 | *N. nucifera* |
| Nn4g24224 | Nn4g24224 | *N. nucifera* |
| Nn4g24225 | Nn4g24225 | *N. nucifera* |
| Nn6g33889 | Nn6g33889 | *N. nucifera* |
| Nn6g35603 | Nn6g35603 | *N. nucifera* |
| Nn6g35607 | Nn6g35607 | *N. nucifera* |
| Nn1g00935 | Nn1g00935 | *N. nucifera* |
| Nn1g00936 | Nn1g00936 | *N. nucifera* |
| Nn1g00941 | Nn1g00941 | *N. nucifera* |
| Nn1g07783 | Nn1g07783 | *N. nucifera* |
| Nn1g07861 | Nn1g07861 | *N. nucifera* |
| Nn1g07921 | Nn1g07921 | *N. nucifera* |
| Nn1g08565 | Nn1g08565 | *N. nucifera* |
| Nn1g08566 | Nn1g08566 | *N. nucifera* |
| Nn1g08575 | Nn1g08575 | *N. nucifera* |
| Nn1g08576 | Nn1g08576 | *N. nucifera* |
| Nn118s43419 | Nn118s43419 | *N. nucifera* |
| Al01214 | Al01214 | *N. lutea* |
| Al01215 | Al01215 | *N. lutea* |
| Al02356 | Al02356 | *N. lutea* |
| Al05222 | Al05222 | *N. lutea* |
| Al08543 | Al08543 | *N. lutea* |
| Al08801 | Al08801 | *N. lutea* |
| Al12619 | Al12619 | *N. lutea* |
| Al12624 | Al12624 | *N. lutea* |
| Al12629 | Al12629 | *N. lutea* |
| Al12940 | Al12940 | *N. lutea* |
| Al15046 | Al15046 | *N. lutea* |
| Al15048 | Al15048 | *N. lutea* |
| Al15050 | Al15050 | *N. lutea* |
| Al19104 | Al19104 | *N. lutea* |
| Al21325 | Al21325 | *N. lutea* |
| Al21347 | Al21347 | *N. lutea* |
| Al21476 | Al21476 | *N. lutea* |
| Al24401 | Al24401 | *N. lutea* |
| Al24404 | Al24404 | *N. lutea* |
| Al24428 | Al24428 | *N. lutea* |
| Al26120 | Al26120 | *N. lutea* |
| Al26309 | Al26309 | *N. lutea* |
| Al27432 | Al27432 | *N. lutea* |
| Al27458 | Al27458 | *N. lutea* |
| Al30082 | Al30082 | *N. lutea* |
| Al30597 | Al30597 | *N. lutea* |
| Al31938 | Al31938 | *N. lutea* |
| Al33381 | Al33381 | *N. lutea* |
| Al33959 | Al33959 | *N. lutea* |
| Al33962 | Al33962 | *N. lutea* |
| Al33964 | Al33964 | *N. lutea* |
| Al35801 | Al35801 | *N. lutea* |

**Table S18. Summary of SNPs from all varieties of lotus**

| **Category** | | **AL** | **WL** | **FL** | **SL** | **RL** | **TL** |
| --- | --- | --- | --- | --- | --- | --- | --- |
| Sample size | | 24 | 21 | 131 | 21 | 30 | 13 |
| Upstream | | 526,757 | 664,676 | 652,409 | 648,619 | 568,487 | 545,857 |
| Exonic | Stop gain | 7,032 | 7,493 | 7,191 | 7,411 | 5,016 | 5,529 |
|  | Stop loss | 1,849 | 2,064 | 2,058 | 1,986 | 1,713 | 1,787 |
|  | Synonymous | 176,240 | 182,527 | 179,236 | 180,426 | 142,190 | 158,656 |
|  | Non-synonymous | 270,705 | 282,887 | 276,987 | 279,570 | 216,261 | 239,720 |
| Intronic | | 3,896,731 | 4,444,561 | 4,197,837 | 4,373,123 | 3,428,623 | 3,582,990 |
| Splicing | | 1,624 | 1,748 | 1,694 | 1,717 | 1,286 | 1,386 |
| Downstream | | 514,227 | 633,919 | 621,339 | 616,374 | 533,272 | 530,592 |
| upstream/downstream | | 39,863 | 48,889 | 47,696 | 47,155 | 40,963 | 40,396 |
| Intergenic | | 7,591,553 | 10,018,397 | 9,744,225 | 9,813,770 | 9,184,263 | 8,561,576 |
| ts |  | 9,311,354 | 11,658,885 | 11,182,541 | 11,411,569 | 10,186,455 | 9,899,063 |
| tv |  | 3,715,735 | 4,628,845 | 4,548,698 | 4,559,145 | 3,936,077 | 3,769,947 |
| ts/tv |  | 2.506 | 2.519 | 2.458 | 2.503 | 2.588 | 2.626 |
| Total |  | 13,027,089 | 16,287,730 | 15,731,239 | 15,970,714 | 14,122,532 | 13,669,010 |

AL, American lotus accessions; WL, wild Asian lotus accessions; FL, flowering lotus accessions; SL, seed lotus accessions; RL, rhizome lotus accessions; TL, Thailand lotus accessions.

**Table S19. Statistics of common and unique SNPs across different subgroups**

| Group Name | Sample size | All_SNP | Unique_SNP | Uniq_Rate (%) | Common_SNP | Common_Rate (%) |
| --- | --- | --- | --- | --- | --- | --- |
| AL | 24 | 13027089 | 434232 | 3.33 | 7174284 | 55.07 |
| WL | 21 | 16287730 | 498516 | 3.06 |  | 44.05 |
| FL | 131 | 15731239 | 385539 | 2.45 |  | 45.61 |
| SL | 21 | 15970714 | 630392 | 3.95 |  | 44.92 |
| RL | 30 | 14122532 | 1157631 | 8.20 |  | 50.80 |
| TL | 13 | 13669010 | 632501 | 4.63 |  | 52.49 |

AL, American lotus accessions; WL, wild Asian lotus accessions; FL, flowering lotus accessions; SL, seed lotus accessions; RL, rhizome lotus accessions; TL, Thailand lotus accessions.

**Table S20. Statistics of Pi and *F*st across different subgroups**

| Group Name | Pi |
| --- | --- |
| AL | 0.001376 |
| TL | 0.001455 |
| WL | 0.000578 |
| FL | 0.004231 |
| SL | 0.000753 |
| RL | 0.000631 |

| Group1_Group2 | *F*st |
| --- | --- |
| WL_FL | 0.140322 |
| WL_RL | 0.112679 |
| WL_SL | 0.668891 |
| FL_RL | 0.15265 |
| FL_SL | 0.137082 |
| RL_SL | 0.675644 |
| AL_WL | 0.9096 |
| TL_FL | 0.040361 |

AL, American lotus accessions; WL, wild Asian lotus accessions; FL, flowering lotus accessions; SL, seed lotus accessions; RL, rhizome lotus accessions; TL, Thailand lotus accessions.

**Table S21. The heterozygosity ratio in each subgroup**

| Group Name | HeteRatio (%) |
| --- | --- |
| AL | 0.198 |
| WL | 0.040 |
| FL | 0.439 |
| SL | 0.090 |
| RL | 0.065 |
| TL | 0.091 |

AL, American lotus accessions; WL, wild Asian lotus accessions; FL, flowering lotus accessions; SL, seed lotus accessions; RL, rhizome lotus accessions; TL, Thailand lotus accessions.

**Table S22. Genes under selection associated with domestication in seed and rhizome lotus accessions**

| Group | Gene ID | | | | | | |
| --- | --- | --- | --- | --- | --- | --- | --- |
| SL | Nn1g00917 | Nn1g00920 | Nn1g00921 | Nn1g00919 | Nn1g00918 | Nn1g00922 | Nn1g00927 |
| selective | Nn1g00964 | Nn1g00977 | Nn1g00990 | Nn1g00989 | Nn1g00991 | Nn1g01007 | Nn1g01003 |
| genes | Nn1g01004 | Nn1g01005 | Nn1g01008 | Nn1g01006 | Nn1g01009 | Nn1g01071 | Nn1g01072 |
|  | Nn1g01073 | Nn1g01078 | Nn1g01079 | Nn1g01081 | Nn1g01080 | Nn1g01275 | Nn1g01526 |
|  | Nn1g01545 | Nn1g01547 | Nn1g01573 | Nn1g01572 | Nn1g01599 | Nn1g01640 | Nn1g01639 |
|  | Nn1g01641 | Nn1g01677 | Nn1g01673 | Nn1g01676 | Nn1g01678 | Nn1g01675 | Nn1g01682 |
|  | Nn1g01787 | Nn1g01785 | Nn1g01786 | Nn1g01843 | Nn1g01847 | Nn1g01845 | Nn1g01848 |
|  | Nn1g01846 | Nn1g01844 | Nn1g01849 | Nn1g01858 | Nn1g01859 | Nn1g01860 | Nn1g04785 |
|  | Nn1g06035 | Nn1g06103 | Nn1g06107 | Nn1g06104 | Nn1g06105 | Nn1g06106 | Nn1g06179 |
|  | Nn1g06177 | Nn1g06178 | Nn1g06176 | Nn1g06180 | Nn1g06452 | Nn1g06453 | Nn1g06543 |
|  | Nn1g06544 | Nn1g06626 | Nn1g08031 | Nn1g08209 | Nn1g08302 | Nn1g08303 | Nn1g08304 |
|  | Nn1g08305 | Nn1g08421 | Nn2g10281 | Nn2g10280 | Nn2g11096 | Nn2g11097 | Nn2g11098 |
|  | Nn2g11106 | Nn2g11108 | Nn2g11109 | Nn2g11107 | Nn2g11114 | Nn2g11115 | Nn2g12065 |
|  | Nn2g12067 | Nn2g12066 | Nn2g12098 | Nn2g12257 | Nn2g12320 | Nn2g12322 | Nn2g12321 |
|  | Nn2g12323 | Nn2g12328 | Nn2g12338 | Nn2g12337 | Nn2g12404 | Nn2g12401 | Nn2g12402 |
|  | Nn2g12403 | Nn2g12422 | Nn2g12421 | Nn2g12423 | Nn3g16670 | Nn3g16669 | Nn3g16681 |
|  | Nn3g16679 | Nn3g16680 | Nn3g16803 | Nn3g16801 | Nn3g16800 | Nn3g16802 | Nn3g16809 |
|  | Nn3g16811 | Nn3g16810 | Nn3g16812 | Nn3g16816 | Nn3g16815 | Nn3g16814 | Nn3g16813 |
|  | Nn3g16817 | Nn3g16879 | Nn3g16880 | Nn3g16877 | Nn3g16878 | Nn3g16876 | Nn3g16881 |
|  | Nn3g17191 | Nn3g17193 | Nn3g17192 | Nn3g17245 | Nn3g17247 | Nn3g17248 | Nn3g17249 |
|  | Nn3g17246 | Nn3g17310 | Nn3g17311 | Nn3g17309 | Nn3g17371 | Nn3g17370 | Nn3g17372 |
|  | Nn3g17423 | Nn3g17421 | Nn3g17422 | Nn3g17424 | Nn3g17425 | Nn3g17426 | Nn3g17427 |
|  | Nn3g17492 | Nn3g17489 | Nn3g17493 | Nn3g17490 | Nn3g17491 | Nn3g17494 | Nn3g17547 |
|  | Nn3g17548 | Nn3g17549 | Nn3g17550 | Nn3g17611 | Nn3g17612 | Nn3g17613 | Nn3g17615 |
|  | Nn3g17614 | Nn3g17830 | Nn3g17831 | Nn3g17832 | Nn3g17833 | Nn3g17838 | Nn3g17839 |
|  | Nn3g17842 | Nn3g17843 | Nn3g17856 | Nn3g17857 | Nn3g17859 | Nn3g17858 | Nn3g17867 |
|  | Nn3g17869 | Nn3g17868 | Nn3g17870 | Nn3g18247 | Nn3g18249 | Nn3g18248 | Nn3g18253 |
|  | Nn3g18250 | Nn3g18252 | Nn3g18251 | Nn3g18795 | Nn3g18797 | Nn3g18794 | Nn3g18796 |
|  | Nn3g18894 | Nn3g18892 | Nn3g18893 | Nn3g18945 | Nn3g18946 | Nn3g18977 | Nn3g18976 |
|  | Nn3g18975 | Nn3g18995 | Nn3g18994 | Nn3g18996 | Nn3g19000 | Nn3g18999 | Nn3g18997 |
|  | Nn3g18998 | Nn3g19022 | Nn3g19021 | Nn3g19024 | Nn3g19025 | Nn3g19032 | Nn3g19034 |
|  | Nn3g19033 | Nn3g19053 | Nn3g19051 | Nn3g19052 | Nn3g19108 | Nn3g19106 | Nn3g19107 |
|  | Nn3g19652 | Nn3g19653 | Nn3g19725 | Nn3g19738 | Nn3g19737 | Nn3g19744 | Nn3g19743 |
|  | Nn3g19742 | Nn3g19795 | Nn3g19811 | Nn3g19810 | Nn3g19861 | Nn3g19862 | Nn3g19867 |
|  | Nn3g19866 | Nn3g19865 | Nn3g19868 | Nn3g20734 | Nn3g21055 | Nn3g21054 | Nn3g21052 |
|  | Nn3g21051 | Nn3g21053 | Nn3g21416 | Nn3g21418 | Nn3g21420 | Nn3g21419 | Nn3g21417 |
|  | Nn3g21702 | Nn3g21699 | Nn3g21700 | Nn3g21701 | Nn4g23804 | Nn4g23805 | Nn4g23803 |
|  | Nn4g23807 | Nn4g23806 | Nn4g23808 | Nn4g23859 | Nn4g23861 | Nn4g23860 | Nn4g24226 |
|  | Nn4g24229 | Nn4g24228 | Nn4g24225 | Nn4g24227 | Nn5g27159 | Nn5g27158 | Nn5g27190 |
|  | Nn5g27197 | Nn5g27198 | Nn5g27275 | Nn5g27277 | Nn5g27276 | Nn5g27278 | Nn5g27279 |
|  | Nn5g27370 | Nn5g27368 | Nn5g27369 | Nn5g27427 | Nn5g27426 | Nn5g27546 | Nn5g27547 |
|  | Nn5g27568 | Nn5g27566 | Nn5g27569 | Nn5g27567 | Nn5g28665 | Nn5g28664 | Nn5g28663 |
|  | Nn5g29584 | Nn5g29585 | Nn5g29708 | Nn5g29710 | Nn5g29707 | Nn5g29709 | Nn5g29711 |
|  | Nn6g31857 | Nn6g31856 | Nn6g31861 | Nn6g31862 | Nn6g31860 | Nn6g31858 | Nn6g31859 |
|  | Nn6g31864 | Nn6g32100 | Nn6g32099 | Nn6g32101 | Nn6g32163 | Nn6g32162 | Nn6g32166 |
|  | Nn6g32164 | Nn6g32165 | Nn6g32167 | Nn6g32168 | Nn6g32169 | Nn6g32171 | Nn6g32170 |
|  | Nn6g32205 | Nn6g32206 | Nn6g32216 | Nn6g32236 | Nn6g32243 | Nn6g32245 | Nn6g32244 |
|  | Nn6g32252 | Nn6g32254 | Nn6g32253 | Nn6g32258 | Nn6g32259 | Nn6g32272 | Nn6g32273 |
|  | Nn6g32274 | Nn6g32275 | Nn6g32291 | Nn6g32294 | Nn6g32292 | Nn6g32293 | Nn6g32314 |
|  | Nn6g32316 | Nn6g32315 | Nn6g32401 | Nn6g33304 | Nn6g33303 | Nn6g33302 | Nn6g33322 |
|  | Nn6g33323 | Nn6g33324 | Nn6g33423 | Nn6g33428 | Nn6g33427 | Nn6g33425 | Nn6g33429 |
|  | Nn6g33426 | Nn6g33430 | Nn6g33526 | Nn6g33560 | Nn6g33558 | Nn6g33559 | Nn6g33566 |
|  | Nn6g33568 | Nn6g33567 | Nn6g33569 | Nn6g33585 | Nn6g33587 | Nn6g33611 | Nn6g33612 |
|  | Nn6g33616 | Nn6g33615 | Nn6g33613 | Nn6g33614 | Nn6g33617 | Nn6g33618 | Nn6g33624 |
|  | Nn6g33623 | Nn6g33622 | Nn6g33625 | Nn7g36748 | Nn7g37647 | Nn7g37646 | Nn7g37648 |
|  | Nn7g37649 | Nn7g37695 | Nn7g37787 | Nn7g37923 | Nn7g38041 | Nn7g38232 | Nn7g38300 |
|  | Nn7g38299 | Nn8g38549 | Nn8g38548 | Nn8g38550 | Nn8g38728 | Nn8g38729 | Nn8g38727 |
|  | Nn8g38799 | Nn8g38798 | Nn8g38800 | Nn8g38803 | Nn8g38801 | Nn8g38802 | Nn8g38887 |
|  | Nn8g38888 | Nn8g38889 | Nn8g38954 | Nn8g38953 | Nn8g38952 | Nn8g38951 | |
| RL | Nn1g00699 | Nn1g00698 | Nn1g01518 | Nn1g01517 | Nn1g01516 | Nn1g01515 | Nn1g02080 |
| selective | Nn1g02081 | Nn1g02402 | Nn1g02398 | Nn1g02399 | Nn1g02401 | Nn1g02403 | Nn1g02400 |
| genes | Nn1g08991 | Nn1g08992 | Nn1g08993 | Nn1g08994 | Nn1g08995 | Nn1g08996 | Nn1g09049 |
|  | Nn1g09051 | Nn1g09048 | Nn1g09050 | Nn1g09047 | Nn1g09232 | Nn1g09233 | Nn1g09231 |
|  | Nn1g09484 | Nn1g09485 | Nn2g10251 | Nn2g10247 | Nn2g10250 | Nn2g10248 | Nn2g10249 |
|  | Nn2g10281 | Nn2g10280 | Nn2g10278 | Nn2g10279 | Nn2g10282 | Nn2g10283 | Nn2g10288 |
|  | Nn2g10289 | Nn2g10287 | Nn2g10284 | Nn2g10286 | Nn2g10285 | Nn2g10301 | Nn2g10302 |
|  | Nn2g10304 | Nn2g10303 | Nn2g10308 | Nn2g10306 | Nn2g10305 | Nn2g10307 | Nn2g11068 |
|  | Nn2g11066 | Nn2g11067 | Nn2g11069 | Nn2g11073 | Nn2g11070 | Nn2g11071 | Nn2g11072 |
|  | Nn2g12664 | Nn2g12663 | Nn2g12665 | Nn2g12666 | Nn2g12670 | Nn2g12671 | Nn2g12991 |
|  | Nn2g12994 | Nn2g12993 | Nn2g12992 | Nn2g13382 | Nn2g13381 | Nn2g13383 | Nn2g13385 |
|  | Nn2g13384 | Nn2g13631 | Nn2g13632 | Nn2g13633 | Nn2g13647 | Nn2g14780 | Nn2g15160 |
|  | Nn2g15158 | Nn2g15159 | Nn2g15216 | Nn2g15218 | Nn2g15217 | Nn2g15271 | Nn2g15272 |
|  | Nn2g15273 | Nn2g15274 | Nn2g15270 | Nn2g15283 | Nn2g15284 | Nn2g15311 | Nn2g15315 |
|  | Nn2g15313 | Nn2g15314 | Nn2g15312 | Nn2g15316 | Nn2g15318 | Nn2g15317 | Nn2g15319 |
|  | Nn2g15339 | Nn2g15340 | Nn2g15342 | Nn2g15341 | Nn2g15358 | Nn2g15359 | Nn2g15390 |
|  | Nn2g15391 | Nn2g15392 | Nn2g15389 | Nn2g15417 | Nn2g15419 | Nn2g15421 | Nn2g15423 |
|  | Nn2g15424 | Nn2g15422 | Nn2g15420 | Nn2g15425 | Nn2g15426 | Nn2g15431 | Nn2g15430 |
|  | Nn2g15429 | Nn2g15432 | Nn2g15434 | Nn2g15435 | Nn2g15433 | Nn2g15572 | Nn2g15571 |
|  | Nn2g15570 | Nn2g15574 | Nn2g15573 | Nn2g15693 | Nn2g15694 | Nn2g15695 | Nn2g15696 |
|  | Nn2g15698 | Nn2g15697 | Nn4g22461 | Nn4g22463 | Nn4g22462 | Nn4g22538 | Nn4g23992 |
|  | Nn4g23993 | Nn4g23995 | Nn4g23991 | Nn4g23994 | Nn4g24046 | Nn4g24044 | Nn4g24047 |
|  | Nn4g24045 | Nn4g24394 | Nn4g24395 | Nn4g24393 | Nn4g24397 | Nn4g24396 | Nn4g24400 |
|  | Nn4g24402 | Nn4g24401 | Nn4g24475 | Nn4g24476 | Nn4g24479 | Nn4g24481 | Nn4g24480 |
|  | Nn4g24482 | Nn4g24483 | Nn4g24487 | Nn4g24497 | Nn4g24498 | Nn4g24495 | Nn4g24496 |
|  | Nn4g24526 | Nn4g24527 | Nn4g24583 | Nn4g24584 | Nn4g24585 | Nn4g24586 | Nn4g24587 |
|  | Nn4g24588 | Nn4g24628 | Nn4g24629 | Nn4g24634 | Nn4g24636 | Nn4g24637 | Nn4g24638 |
|  | Nn4g24635 | Nn4g24633 | Nn4g24639 | Nn4g24640 | Nn4g24644 | Nn4g24643 | Nn4g24642 |
|  | Nn4g24641 | Nn4g24645 | Nn4g24648 | Nn4g24647 | Nn4g24646 | Nn4g24649 | Nn4g24763 |
|  | Nn4g24761 | Nn4g24764 | Nn4g24762 | Nn4g24765 | Nn4g24770 | Nn4g24771 | Nn4g24827 |
|  | Nn4g24828 | Nn4g24829 | Nn4g24830 | Nn4g24843 | Nn4g24844 | Nn4g24862 | Nn4g24866 |
|  | Nn4g24864 | Nn4g24865 | Nn4g24919 | Nn4g24920 | Nn4g24921 | Nn4g25116 | Nn4g25118 |
|  | Nn4g25117 | Nn4g25119 | Nn4g25120 | Nn4g25885 | Nn4g25997 | Nn4g25998 | Nn4g26000 |
|  | Nn4g25999 | Nn4g26424 | Nn4g26425 | Nn5g26779 | Nn5g26783 | Nn5g26781 | Nn5g26784 |
|  | Nn5g26782 | Nn5g26780 | Nn5g26785 | Nn5g26979 | Nn5g26978 | Nn5g26980 | Nn5g26981 |
|  | Nn5g27149 | Nn5g27150 | Nn5g28626 | Nn5g28627 | Nn5g28628 | Nn5g28629 | Nn5g28630 |
|  | Nn5g28632 | Nn5g28631 | Nn6g31772 | Nn6g31774 | Nn6g31771 | Nn6g31773 | Nn6g31770 |
|  | Nn6g31777 | Nn6g31776 | Nn6g31775 | Nn6g31849 | Nn6g31850 | Nn6g31851 | Nn6g31852 |
|  | Nn6g32014 | Nn6g32013 | Nn6g32251 | Nn6g32252 | Nn6g32258 | Nn6g32260 | Nn6g32309 |
|  | Nn6g32381 | Nn6g33351 | Nn6g33352 | Nn6g33365 | Nn6g33366 | Nn6g33367 | Nn6g33377 |
|  | Nn6g33378 | Nn6g33727 | Nn6g33728 | Nn6g33729 | Nn6g33732 | Nn6g33731 | Nn6g33730 |
|  | Nn6g33747 | Nn6g33746 | Nn6g33824 | Nn6g34312 | Nn6g34315 | Nn6g34314 | Nn6g34313 |
|  | Nn7g36074 | Nn7g36800 | Nn7g36803 | Nn7g36801 | Nn7g36802 | Nn7g37064 | Nn7g37062 |
|  | Nn7g37063 | Nn7g37066 | Nn7g37065 | Nn7g37373 | Nn7g37393 | Nn7g37392 | Nn7g37396 |
|  | Nn7g37395 | Nn7g37397 | Nn7g37421 | Nn7g37422 | Nn7g37451 | Nn7g37450 | Nn7g37789 |
|  | Nn7g38080 | Nn7g38081 | Nn8g39938 | Nn8g39940 | Nn8g39941 | Nn8g40382 | |

**Table S23. List of primers used for qRT-PCR in this study**

| **Gene** | **Gene ID** | **Forward Primer (5'-3')** | **Reverse Primer (5'-3')2** |
| --- | --- | --- | --- |
| εLCY | Nn8g38788 | AGACCAAGCCCATGGACAAG | AATTCGTCCTCCCACACACC |
| βLCY | Nn7g36588 | TGGCCCATAGAGAGGAGGAG | AGAGGAAGAGTGCCCTTTGC |
| PDS | Nn6g31492 | AGGGGAGTTCAGCCGATTTG | AACATAAGCCTGTCCGCCAA |
| ZDS | Nn1g02298 | CTGTTGCTTATGCCCTGGGA | ATCTCTCTGCATCCCCACCT |
| ZEP | Nn5g30293 | CCATATTTGGGTGTGGGGCT | TGTGAGTCTGCAGCAGAGTG |
| Actin | Nn7g36007 | GGATTTGCCGGTGATGATGC | GTCATCCCAGTTGCTGACGA |
| PSY | Nn4g24773 | ATGCCAGCTGCTACACTGTT | CCATCTGGGTTTCTTCCCCC |
| NCED3 | Nn3g20474 | GCTCTGCTCCGCAATGATTG | TCAACGTCCGGTGACTTCTG |
| NSY | Nn3g17869 | GGACACCTGACACCATACGG | GTGCCGGGTCTCAATCTCAT |
| CISO | Nn2g14414 | GGAGAGAGGTGGGAGTGGAT | TAGTACCCTGAGCTCCCACC |
| Z-ISO | Nn1g09788 | TGAATTCGCGAGACCCACAA | GCGAAGAGAACAGTCCCCAA |
| VDE | Nn1g07946 | ATGTTGTGGCTGCAGCTAGT | GCAATGTTGGCTGCACATGA |
| MYB5 | Nn4g24223 | ATGGATGGTGGTTTGGGTTT | TCAATAACTCCACCACCTATG |
| F3'H | Nn3g17431 | TGGACGAACACAGGGTCATG | TGTCGGTGCCTGCTGTAAAT |
| ANGT | Nn8g40696 | TACGGCAAAGTCGAACTGCT | AACTGCCTTGAAGTTGCCCT |
| ANS | Nn1g01275 | GGCTCAACCACTTGCAGTTG | AGCTCTTCTTGAGGACGCAC |
| F3H | Nn5g28145 | ATCGACACCATCCCTCCTGA | TTGACGACCTGGAAGATGCC |
| CHS | Nn3g19912 | CTGTGTCGACCAGAGCACAT | CTCCTCGGTCAAGTGCATGT |
| FLS | Nn5g30144 | CATTCGCCGAGATGTACCGA | TTTTGCTTTCCGCTTCCAGC |
| F3'5'H | Nn7g36756 | AGCATTCTGAGACGTGCACA | CATTCACCTCGCATGCTTCG |
| CHI | Nn2g10310 | TAAGTGGAAGCTGTGGCCTG | ATCACGGTTTCGGTGCTCAT |
| DFR | Nn5g28645 | GAACACCAACAGCCCGAGTA | ATTCCCAGGCTGCTTTCTCC |
